# Toward evidence-based prescription of prosthetic ankle-foot devices: A multisite randomized crossover trial identifying performance-based, patient-reported, and biomechanical parameters sensitive to device type

**DOI:** 10.1371/journal.pone.0352644

**Published:** 2026-07-02

**Authors:** Jason T. Maikos, Alexis N. Sidiropoulos, John M. Chomack, David V. Herlihy, Brad D. Hendershot, Alison L. Pruziner, Samuel L. Phillips, Jeffrey T. Heckman, Timothy E. Moore, Zoe Gibbs McBride, Jongwoo Choi, Christopher L. Dearth, The Prosthetic Foot Prescription Consortia, Leif M. Nelson

**Affiliations:** 1 Prosthetics and Sensory Aids Service, Veterans Affairs New York Harbor Healthcare System, New York, New York, United States of America; 2 Narrows Institute for Biomedical Research and Education, Inc., Brooklyn, New York, United States of America; 3 Department of Rehabilitation, Walter Reed National Military Medical Center, Bethesda, Maryland, United States of America; 4 Extremity Trauma and Amputation Center of Excellence, Defense Health Agency, Falls Church, Virginia, United States of America; 5 Department of Physical Medicine & Rehabilitation, Uniformed Services University of the Health Sciences, Bethesda, Maryland, United States of America; 6 Department of Veterans Affairs, National Veterans Sports Programs and Special Events, Washington, District of Columbia, United States of America; 7 Physical Medicine & Rehabilitation Service, James A. Haley Veterans’ Hospital and Clinics, Tampa, Florida, United States of America; 8 Division of Rehabilitation Care Services, Veterans Affairs Puget Sound Healthcare System, Seattle, Washington, United States of America; 9 Department of Rehabilitation Medicine, University of Washington, Seattle, Washington, United States of America; 10 Statistical Consulting Services, Center for Open Research Resources and Equipment, University of Connecticut, Storrs, Connecticut, United States of America; 11 Department of Statistics, University of Connecticut, Storrs, Connecticut, United States of America; Roma Tre University: Universita degli Studi Roma Tre, ITALY

## Abstract

Prescription of prosthetic ankle-foot devices is constrained by imprecise clinical guidelines and inconsistent scientific evidence, hindering optimal device selection for individuals with lower limb loss. This multisite, prospective, randomized crossover study aimed to identify patient-reported, performance-based, and biomechanical parameters sensitive to ankle-foot device type, providing a foundation for more objective and individualized prescription practices. Ninety-one individuals with unilateral transtibial limb loss completed the crossover trial, and 13 control participants without musculoskeletal impairment were enrolled to provide normative reference data. Participants were fitted with duplicate sockets and randomized to trial three ankle-foot device types: energy storing and returning, articulating, and powered. Participants were heterogeneous in demographic characteristics, including veterans, service members, and civilians. After one week of acclimation per device, participants completed performance-based (6-minute walk, Timed Up and Go, Four Square Step Test, Stair and Hill Assessment Indices, Amputee Mobility Predictor) and patient-reported (Prosthesis Evaluation Questionnaire, 12-Item Short Form Health Survey, Orthotics and Prosthetics Users’ Survey) assessments; a subset (n = 29 completed) underwent full-body gait analysis to capture detailed biomechanical parameters. Biomechanical outcomes demonstrated the greatest sensitivity to device type, with 19 distinct parameters, primarily at the ankle, highlighting ankle mechanics as a key determinant of differences among prosthetic devices. Five Prosthesis Evaluation Questionnaire subscales were sensitive to device type, while performance-based measures showed no significant effects. Results revealed a dichotomy between biomechanical and patient-reported outcomes: Biomechanical parameters were more similar to control values for powered devices, whereas patient-reported outcomes favored non-powered devices. Linear discriminant analysis identified key gait features, including peak plantarflexion during preswing and peak ankle moment, which most strongly contributed to group separation and clinical discrimination. These findings identified distinct biomechanical and patient-reported parameters sensitive to ankle-foot device type and highlight the need for evidence-based, individualized prosthetic prescription to optimize device selection and improve patient outcomes.

## Introduction

As of 2019, approximately 2.3 million Americans were living with limb loss [1]. As this already large population continues to grow and age, the need for effective, outcomes-based clinical practice will become increasingly critical to reduce long-term disability and improve quality of life. For individuals with transtibial limb loss, providers within the limb loss care team can select from more than 300 commercially available ankle-foot devices [2]. While an effective prosthetic prescription is essential for successful rehabilitation following limb loss, the process remains complex and challenging, particularly as prosthetic ankle-foot devices have evolved over the last two decades [3]. Current device selection is largely governed by professional judgment of the care team, guided by patient evaluation, interviews assessing current abilities and goals, and previous prosthesis use [4]. However, this approach can be influenced by individual provider biases and constrained by an inconsistent evidence base that tends to be noncommittal or lacks the guidance required for clinical practice [5].

Efforts to establish scientific or consensus-based prosthetic prescription have been hindered by small sample sizes, heterogeneous methodologies, and contradictory findings [6–12]. Clinical consensus, informed by the expertise of the multidisciplinary limb loss care team, has attempted to identify critical parameters and predictors of lower limb prosthetic prescription [8]. However, these recommendations are rooted in a combination of anecdotal perspectives and contradictory research outcomes in the available scientific literature, limiting broad applicability. Less commonly, investigators have attempted to develop prescription guidelines by matching device mechanical behavior with individual patient characteristics [13]. However, there are no data that correlate individual patient characteristics to different mechanical properties of ankle-foot devices. Similarly, studies comparing ankle-foot devices within a single mechanical category have not consistently identified clear biomechanical patterns [14,15]. Most commonly, comparative effectiveness research evaluating patient-reported, performance-based, bioenergetic, and biomechanical outcomes has aimed to strengthen the empirical evidence base [16–18], but has often produced inconclusive or non-generalizable findings that preclude the direct application of scientific evidence to clinical decision making [6,7,17,19]. Consequently, clinicians still lack reliable predictors of how specific device characteristics influence biomechanical and functional performance.

Although several valid and reliable assessment tools exist for assessing prosthetic performance, no “gold standard” measures exist to guide device selection across the diverse needs, abilities, and goals of prosthesis users [6,9,19–21]. Identifying outcome measures that are sensitive to differences among prosthetic ankle-foot devices is therefore a critical foundational step toward evidence-based prescription.

To address these gaps, this multicenter clinical trial enrolled a large and diverse sample of veterans, service members, and civilians to evaluate representative categories of prosthetic ankle-foot devices. The study aimed to identify patient-reported, performance-based, and biomechanical parameters sensitive to ankle-foot type across multiple domains of prosthetic performance, providing a foundation for developing more objective and individualized prosthetic prescription practices. Specifically, this study compared three broad categories of ankle-foot devices, including energy storing and returning (ESR), articulating ESR (ART), and powered ESR (PWR) devices, to establish benchmark measures for future clinical applications.

## Materials and methods

### Study design

This prospective, multicenter investigation was conducted across four sites: Veterans Affairs New York Harbor Healthcare System (VANYHHS), Veterans Affairs Puget Sound Healthcare System, James A. Haley Veterans’ Hospital, and Walter Reed National Military Medical Center (WRNMMC). Sample size was determined a priori using power analyses of functional, patient-reported, and biomechanical outcomes, based on pilot data and assuming an α level of 0.05 with within-participant standard deviations; full details are provided in the published protocol [22]. Participants were adults (>18 years) with unilateral transtibial limb loss who were current prosthesis users with a well-fitting socket and scored at least modified independence on the Functional Independence Measure. Full eligibility criteria are reported in the published protocol [22]. A total of 106 individuals with unilateral transtibial limb loss were enrolled, of whom 91 completed the study. Participants completed a randomized crossover trial, using each of the three ankle-foot device types with duplicate sockets and device-specific training, with a 1-week acclimation period for each device. Additionally, a control cohort of 13 individuals without musculoskeletal impairment was added via an approved protocol amendment during the study period to collect normative biomechanical, performance-based, and patient-reported data for comparison. The addition of this cohort did not alter the randomized crossover intervention or study procedures for participants with limb loss, and analyses were conducted separately to provide reference values.

**Fig 1:**
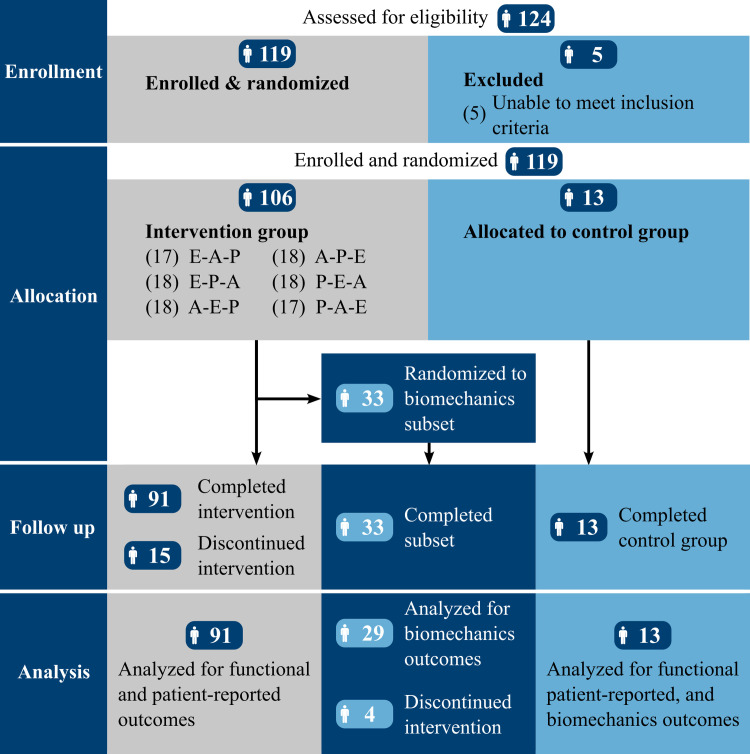
Participant flow through the randomized crossover ankle-foot device trial. CONSORT flow diagram illustrating participant screening, enrollment, allocation to intervention sequences, completion of crossover testing, and analysis populations. A nested biomechanics subgroup and parallel control group are also shown. **Abbreviations**: *E: energy storing and returning; A: articulating energy storing and returning; P: powered energy storing and returning*.

At study initiation, a multidisciplinary expert panel systematically selected and categorized commercially available devices by structure, componentry, performance-based characteristics, and biomechanical properties into three categories: Non-articulating ESR (29 included devices), ART (13 included devices), and PWR (one included device: Ottobock Empower), as previously described [22]. Participants (87%) used their previously prescribed ESR device when available, with the remainder randomly assigned a commercially available ESR option, which was a pragmatic approach aimed at reflecting real-world prescription practices. ART devices were block randomized using a computer-generated allocation sequence implemented centrally, with study staff who enrolled participants blinded to the sequence until assignments were made; device order was further randomized to minimize order effects. The same PWR device was used for all participants. Data processors were blinded to device type, with data coded to mask the device assignment. Participants and prosthetists were not blinded, due to the distinct mechanical and aesthetic characteristics of the devices. The trial was registered at ClinicalTrials.gov (NCT03505983). Participant recruitment occurred at each site as follows: VANYHHS (April 24, 2018 to February 17, 2023), James A. Haley Veterans’ Hospital (May 15, 2018 to February 14, 2023), Veterans Affairs Puget Sound Healthcare System (May 24, 2018 to February 27, 2020), and WRNMMC (June 11, 2018 to April 6, 2022). Final follow-up was completed on September 14, 2023. Participant screening, enrollment, allocation to intervention sequences, and analysis populations are summarized in the Consolidated Standards of Reporting Trials (CONSORT) flow diagram (Fig 1). The study was conducted in accordance with the approved protocol [22], which is provided as Supporting Information (S1 Protocol).

Following written informed consent, demographic data were collected, including age, sex, race, ethnicity, height, body mass, limb loss etiology, employment, marital status, living situation, time since limb loss, and clinician-derived Medicare Functional Classification System category (K-level). Participants used each device for a 1-week acclimation period, after which performance-based and patient-reported assessments were performed. A randomly selected subset of participants (n = 33) was assigned to undergo full gait analysis after each acclimation period to collect biomechanical data during level ground walking using each ankle-foot device; 29 participants completed the biomechanical testing. This study was approved by the VANYHHS Institutional Review Board (IRB) (protocol ID 1603), the University of South Florida IRB as the IRB of record for the James A. Haley Veterans’ Hospital (IRB study Pro00030555), the WRNMMC IRB (protocol ID WRNMMC-2017-0103), and the Veterans Affairs Puget Sound Healthcare System IRB (protocol ID 01587). All participants provided written informed consent prior to participation. Adverse events were monitored throughout device acclimation and testing sessions, and captured through participant self-report, clinician observation, and review of the electronic medical record.

### Patient and public involvement

Patients and members of the public were not formally involved in the design, conduct, reporting, or dissemination of this study. However, clinicians were involved in the study design, and patient observations during routine care informed outcome selection and protocol refinement.

### Socket duplication, fitting, and training

Participants’ existing sockets were evaluated for fit and comfort by the study prosthetist at each site using standardized prosthetic guidelines. The well-fitting socket was duplicated twice using identical materials and componentry to reduce variability. Each duplicate socket was bench and dynamically aligned with each assigned ankle-foot device following manufacturer specifications. Participants then received device-specific training from the study physical therapist and were required to achieve a Functional Independence Measure score ≥ 6 (modified independence) for level walking and stair negotiation prior to testing.

### Performance-based and patient-reported assessments

Performance-based and patient-reported measures were designated as the primary outcomes of this study, reflecting clinically relevant indicators of functional mobility and user experience with each ankle-foot device. After each acclimation period, participants completed a standardized battery of six performance-based outcome measures (6-minute walk test, Timed Up and Go (TUG), Four Square Step Test, Amputee Mobility Predictor, Stair Assessment Index, and Hill Assessment Index) and three patient-reported surveys (Prosthesis Evaluation Questionnaire (PEQ), 12-Item Short Form Health Survey (SF-12), and Orthotics and Prosthetics Users’ Survey (OPUS)). A final exit interview captured overall device preference.

### Biomechanical assessment

Biomechanical gait parameters obtained from motion analysis were designated as secondary outcomes to provide mechanistic insight into device-specific effects on lower-limb gait mechanics. The subset of participants who completed biomechanical testing (n = 29) underwent full-body gait analysis after each acclimation period to collect biomechanical data during level ground walking at VANYHHS or WRNMMC. The motion capture equipment and laboratory space at each site have been previously described [22]. Marker trajectories were recorded at 120 Hz and ground reaction forces at 1,200 Hz. A custom marker set of 78 passive reflective markers was placed or digitized on the head, trunk, pelvis, and extremities [22]. Markers on the prosthetic limb mirrored the intact limb or were positioned on device centers of rotation. Functional joint centers were calculated for the hips, knees, and intact ankle [23]. Participants walked at three controlled speeds (1.0, 1.3, and 1.5 m/s) across instrumented walkways (4 m at VANYHHS; 10 m at WRNMMC). Five successful steps per limb were collected at each speed, defined by full foot contact with a force platform. Walking speed order was randomized, and auditory feedback maintained the target speed within ±7%.

### Biomechanical data analysis

**Fig 2:**
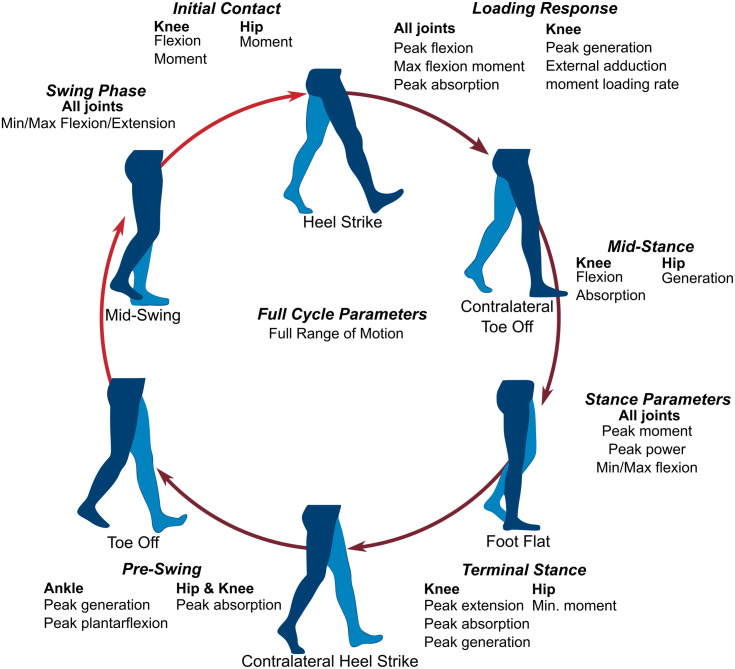
Biomechanical parameters analyzed across lower limb joints. Schematic illustration of the associated biomechanical parameters evaluated in this study. Parameters are organized by gait phase and joint, including ankle, knee, and hip kinematics and kinetics, such as joint angles, moments, power generation and absorption, and range of motion. **Abbreviations:** *Min: minimum; Max: maximum.*

A 15-segment rigid body model was created for each participant following International Society of Biomechanics recommendations [24,25]. Marker trajectories were filtered using a 6 Hz low-pass Butterworth filter. Force plate signals were filtered using a second-order low-pass Butterworth filter with a 25 Hz cutoff frequency. Visual3D (HAS Motion, Inc.) computed temporal-spatial parameters, kinematics, and kinetics. Inverse dynamic analysis was applied to the kinematic data and to the location, magnitude, and direction of ground reaction forces acting on the foot to calculate lower limb joint torques and powers for the intact and prosthetic limbs during stance. Temporal-spatial and discrete biomechanical parameters (previously described in [22]) were extracted for each ankle-foot device type during relevant phases of the gait cycle (Fig 2). For each parameter, mean, maximum and minimum values, ranges of motion, standard errors (SEs), and coefficients of variation were calculated across gait cycles for each condition.

### Statistical analyses

For each performance-based, patient-reported, and biomechanical outcome measure, descriptive statistics (means ± SE) were calculated across ankle-foot device categories. To identify outcome measures most sensitive to changes in ankle-foot device type, linear mixed-effects models were fit with participant characteristics included as covariates; fixed effects were K-level, race, ethnicity, veteran status, height, body mass, limb loss etiology, time since initial limb loss, employment status, living situation, marital status, and age. A participant-specific random intercept was included to account for repeated measures. Model assumptions included independence, linearity of the response in the covariates, and normally distributed, homoscedastic residuals. All randomized participants with available outcome data were included in the analyses of performance-based and patient-reported outcomes. The biomechanical subset included only participants who completed gait testing (n = 29). Missing data were handled using the mixed-effects modeling approach, which incorporates all available observations without imputation. Separate models were fit for each biomechanical, performance-based, and patient-reported measure at each walking speed.

Pairwise comparisons of marginal means were conducted to assess differences among device types. Specifically, the mixed-effects model was fit and then used to predict the biomechanical, performance-based, or patient-reported measure of interest for each ankle-foot type, with all continuous covariates set to their average values and all other factor covariates weighted by their frequency of appearance in the data set. Using these fitted values, the difference in response for each unique pair of ankle-foot device types was estimated, as well as the variance associated with these estimates. Hypothesis tests were performed with the null hypothesis indicating no difference between the ankle-foot device types and the alternative hypothesis indicating a difference between ankle-foot types. P-values were adjusted for multiple comparisons using Tukey’s Honestly Significant Difference. Biomechanical parameters with significant pairwise differences were compared with control data to compute percent change and rank devices from closest to furthest from control values.

Linear discriminant analysis (LDA) was performed for dimensionality reduction and to identify biomechanical parameters that maximized between-device type variance while minimizing within-device type variance to define the most important, discriminative, and predictive gait-related features [26]. With three device categories, data were projected onto a two-dimensional space using two discriminant functions (LD1 and LD2). LDA was fit separately for the ankle, knee, and hip, using device type as the grouping factor and joint-specific biomechanical parameters as predictors. Data were pooled across walking speeds, predictors were standardized, and analyses were performed using all available observations. Group separation was assessed via the proportion of trace explained by LD1 and LD2, and parameter importance by the magnitude of the standardized LDA loadings.

## Results

### Participant characteristics

A total of 124 individuals were assessed for eligibility, including 111 participants with unilateral transtibial limb loss for the intervention and 13 individuals without musculoskeletal impairment for the control cohort. Five participants with transtibial limb loss were excluded prior to enrollment due to not meeting inclusion criteria. Of the 106 participants enrolled, 91 participants with unilateral transtibial limb loss completed all study procedures (Table 1), while 15 participants withdrew for personal reasons. All 13 control participants completed study procedures. No serious adverse events related to device use or study procedures were observed.

**Table 1:** Demographics for all participants.

	Sample Population (n = 91)	Control Population (n = 13)
**Age (years)**		
Mean (SD)	59.6 (12.6)	38.2 (11.9)
**Height (cm)**		
Mean (SD)	179.5 (7.7)	175.6 (9.3)
**Body Mass (kg)**		
Mean (SD)	94.8 (22.0)	83.6 (10.5)
**Sex**		
Men n (%)	83 (91.2)	11 (84.6)
Women n (%)	8 (8.8)	2 (15.4)
**Etiology of Limb Loss**		
Trauma n (%)	51 (56.0)	
Vascular Disease/Diabetes n (%)	33 (36.3)	
Cancer n (%)	5 (5.5)	N/A
Other n (%)	2 (2.2)	
**Time Since Limb Loss (months)**		
Mean (SD)	99.5 (128.3)	N/A
**Clinician-derived K-Level**		
K2 n (%)	3 (3.3)	
K3 n (%)	57 (62.6)	N/A
K4 n (%)	31 (34.1)	
**Suspension Type**		
Suction n (%)	44 (48.4)	
Pin-Locking n (%)	43 (47.3)	N/A
Elevated Vacuum n (%)	4 (4.4)	
**Ethnicity**		
Not Hispanic or Latino n (%)	80 (87.9)	13 (100)
Hispanic or Latino n (%)	7 (7.7)	0 (0)
Not Reported n (%)	4 (4.4)	0 (0)
**Race**		
White n (%)	60 (65.9)	11 (84.6)
Black or African American n (%)	23 (25.3)	0 (0)
American Indian or Native Alaskan n (%)	2 (2.2)	0 (0)
Identified as more than 1 race n (%)	5 (5.5)	0 (0)
Asian n (%)	1 (1.1)	2 (15.4)
**Military Status**		
Veteran n (%)	68 (74.7)	0 (0)
Civilian n (%)	16 (17.6)	3 (23.1)
Active-Duty Service Member n (%)	7 (7.7)	10 (76.9)
**Employment Status**		
Not currently employed n (%)	54 (59.3)	0 (0)
Full time n (%)	26 (28.6)	13 (100)
Part time n (%)	7 (7.7)	0 (0)
Other n (%)	4 (4.4)	0 (0)
**Living Situation**		
Lives with others n (%)	72 (79.1)	11 (84.6)
Lives alone n (%)	19 (20.9)	2 (15.4)
**Relationship Status**		
Married n (%)	46 (50.5)	11 (84.6)
Divorced n (%)	25 (27.5)	0 (0)
Never Married n (%)	12 (13.2)	2 (15.4)
Separated n (%)	5 (5.5)	0 (0)
Widowed n (%)	3 (3.3)	0 (0)

### Performance-based outcomes

**Fig 3:**
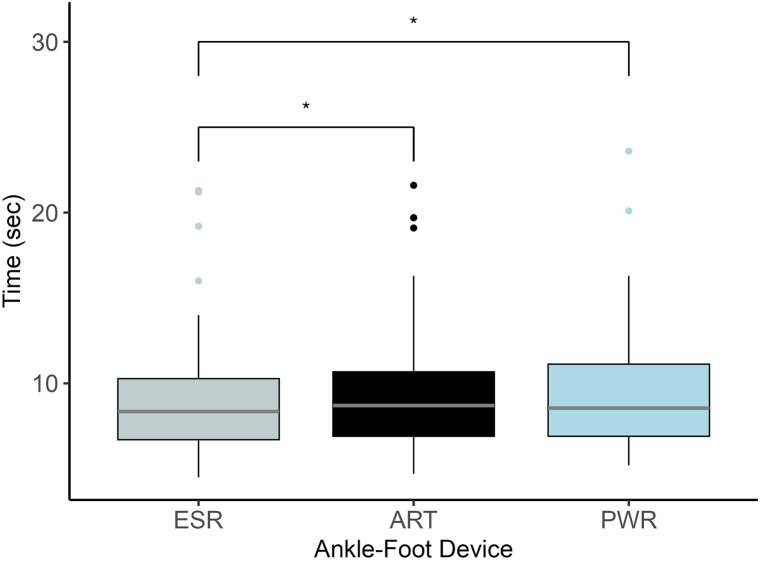
Timed Up and Go performance across ankle-foot device types. Timed Up and Go completion times are shown for energy storing and returning, articulating, and powered ankle-foot devices. Completion times were significantly faster with energy storing and returning devices compared with the articulating and powered devices, though the mean differences did not exceed the minimal detectable change threshold. **Abbreviations:** *ESR: energy storing and returning; ART: articulating energy storing and returning; PWR: powered energy storing and returning.*

Means and standard deviations for all performance-based outcome measures are provided in S1 Table. Linear mixed-effects modeling indicated that TUG (Fig 3) was the only performance-based outcome measure significantly associated with device type. TUG times were faster for ESR compared with ART (p = 0.004) and PWR (p = 0.037); however, the 0.4 s difference did not exceed the minimal detectable change threshold of 3.6 seconds [27]. No other performance-based measures were sensitive to device type.

### Patient-reported outcomes

**Fig 4:**
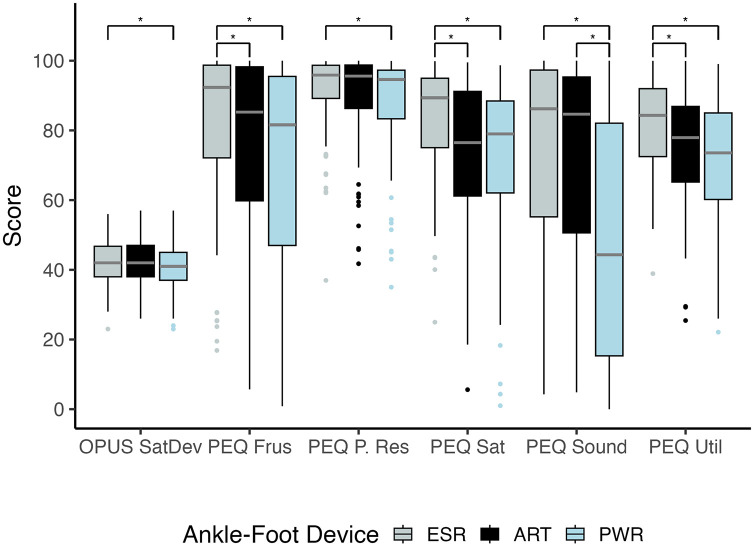
Patient-reported outcome measures showing significant differences across ankle-foot device types. Scores for Prosthesis Evaluation Questionnaire (PEQ) domains and the Orthotics and Prosthetics Users’ Survey (OPUS) Satisfaction with Devices domain are shown for energy storing and returning, articulating, and powered ankle-foot devices. Five PEQ and one OPUS domain were sensitive to device type, though the minimal detectable change threshold was not exceeded for OPUS Satisfaction with Devices. **Abbreviations**: *OPUS: Orthotics and Prosthetics Users’ Survey; PEQ: Prosthesis Evaluation Questionnaire; SatDev: Satisfaction with Devices; Frus: Frustration;* *P.* *Res: Perceived Response; Sat: Satisfaction; Util: Utility; ESR: energy storing and returning; ART: articulating energy storing and returning; PWR: powered energy storing and returning.*

Means and standard deviations for all patient-reported measures are provided in S2 Table. Six measures demonstrated significant effects of device type (Fig 4). ESR scored higher than ART and PWR for PEQ satisfaction (p = 0.002, p < 0.001), PEQ frustration (p = 0.035, p = 0.001), and PEQ utility (p = 0.003, p < 0.001). ESR also scored higher than PWR for PEQ perceived response (p = 0.007) and OPUS satisfaction with devices (p = 0.041). PWR scored lower than ESR and ART devices for PEQ sounds (both p < 0.001). Minimal detectable change thresholds were exceeded for all significant PEQ scores, but not for OPUS Satisfaction with Devices [16]. In exit interviews, participants most frequently preferred ESR (41%), followed by ART (38%), and PWR (21%).

### Discrete biomechanical outcomes

Demographic information for completed participants in the gait analysis subset is presented in S3 Table. Of the 49 unique biomechanical parameters analyzed, 19 were sensitive to device type: 11 at the ankle, six at the knee, and two at the hip. Across the three walking speeds, 85 significant pairwise comparisons were identified (S4 Table). For each significant parameter, percent change from normative control values was calculated and used to rank devices from closest to furthest from controls. The PWR device most frequently demonstrated values closest to controls (20 parameters), followed by ESR (10 parameters) and ART (9 parameters). Ankle-level parameters accounted for the largest proportion of sensitive parameters, while no device type demonstrated a clear advantage for hip-level parameters.

### Linear discriminant analysis

**Fig 5:**
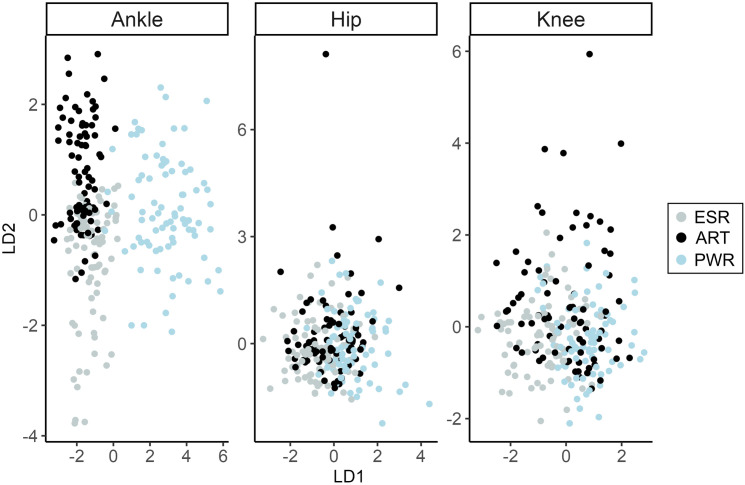
Linear discriminant analysis of biomechanical parameters for each joint by ankle-foot device type. Separate linear discriminant analyses (LDAs) were performed at the ankle, knee, and hip to identify biomechanical parameters that differentiated energy storing and returning (ESR), articulating (ART), and powered (PWR) ankle-foot devices. Ankle-level LDA results demonstrated separation of the PWR device from the ESR and ART devices, with minimal separation between ESR and ART. The first linear discriminant (LD1) accounted for 91.0% of the between-device variance, while the second linear discriminant (LD2) accounted for 9.0%. In contrast, LDAs at the knee and hip showed substantial overlap among device types, indicating limited discriminatory capability at those joints. **Abbreviations:** *LDA*: *linear discriminant analysis; LD1: first linear discriminant; LD2: second linear discriminant;* ESR: energy storing and returning; ART: articulating energy storing and returning; PWR: powered energy storing and returning.

Separate LDAs were performed for the ankle, knee, and hip to identify biomechanical parameters that most strongly differentiated ankle-foot device types. Parameters with the largest standardized coefficients indicated the greatest influence on discrimination. As shown in Fig 5, ankle-level LDA indicated excellent separation of the PWR device from the ESR and ART devices, but minimal separation between ESR and ART devices. LD1 accounted for 91.0% of the variation among device types, whereas LD2 explained 9.0% of the variation. In contrast, LDAs at the knee and hip showed a high degree of overlap, indicating limited discriminatory capability.

**Fig 6:**
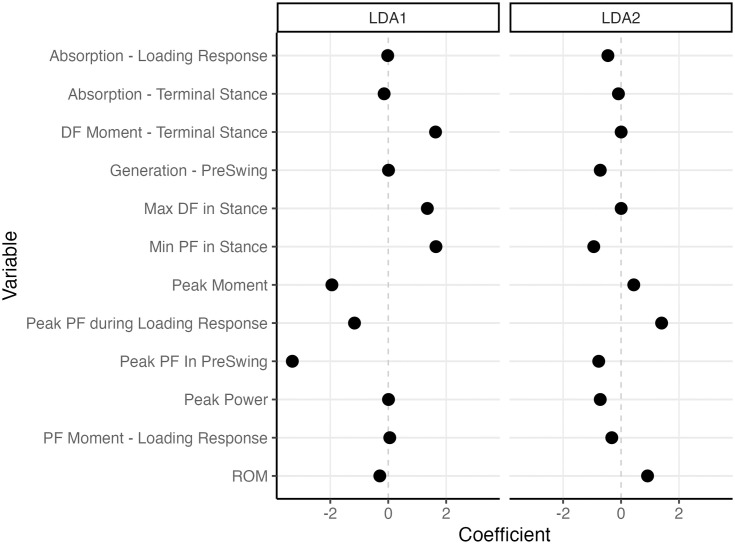
Standardized coefficients from ankle-level linear discriminant analysis. Standardized coefficients with the largest absolute magnitudes are shown for the ankle-level linear discriminant analysis. For linear discriminant 1 (LD1), the primary contributing parameters included peak plantarflexion during preswing, peak ankle moment, minimum plantarflexion in stance, dorsiflexion moment in terminal stance, maximum dorsiflexion in stance, and peak plantarflexion during loading response. For linear discriminant 2 (LD2), the largest contributing parameters included peak plantarflexion during loading response, minimum plantarflexion in stance, ankle range of motion, peak plantarflexion during preswing, ankle power generation in preswing, and peak ankle power. **Abbreviations:** *DF: dorsiflexion; LD1: linear discriminant 1; LD2: linear discriminant 2; Max: maximum; Min: minimum; PF: plantarflexion; ROM: range of motion.*

After reducing dimensionality, the coefficients with the highest absolute magnitudes from the ankle were identified for each discriminant function (Fig 6). For LD1, the primary contributors included peak plantarflexion during preswing, peak ankle moment, minimum plantarflexion in stance, dorsiflexion moment in terminal stance, maximum dorsiflexion in stance, and peak plantarflexion during loading response, in that order. LD2 was primarily influenced by peak plantarflexion during loading response, minimum plantarflexion in stance, ankle range of motion, peak plantarflexion in preswing, ankle power generation in preswing, and peak ankle power. Collectively, these findings underscore the discriminative value of ankle-level gait parameters in differentiating among ESR, ART, and PWR devices, while biomechanical measures had a weaker ability to discriminate ankle-foot types at the knee or the hip.

## Discussion

The current standard of care for prescription of prosthetic ankle-foot devices is hampered by imprecise clinical guidelines and a lack of consistent scientific evidence [7,17]. This randomized multisite study identified a preliminary set of distinct patient-reported and biomechanical parameters sensitive to ankle-foot device type, establishing a foundation for evidence-based prescription. The parameters assessed in this study were selected based on their routine use in research studies or clinical practice [28–30], prior identification as relevant to individuals with lower limb loss [31], and established validity and reliability in this population [32–36]. This study included a large, heterogeneous sample with respect to demographic and background characteristics, enrolling veterans, service members, and civilians, and examined a broad range of ankle-foot devices categorized by shared characteristics [22] to develop this foundational information. Although participants were generally high functioning and in good health, the diversity in age, service status, and life experience supports generalizability of findings to comparable clinical populations.

As expected, biomechanical assessments yielded the highest number of parameters sensitive to device type, in part due to the large number of parameters that were evaluated. Patient-reported outcome measures demonstrated the next highest number of sensitive parameters, while only one performance-based outcome measure, TUG, showed sensitivity to device type. However, the observed differences in mean TUG scores did not exceed the minimal detectable change threshold [27]. A notable divergence emerged between biomechanical and patient-reported outcomes: Biomechanical outcomes for the PWR device tended to be more similar to control values, particularly for ankle-specific parameters, whereas most patient-reported outcomes had higher scores for the ESR and ART devices. This dichotomy is explored in greater detail in the sections that follow.

### Performance-based outcome measures

Performance-based outcome measures are commonly used in clinical practice and limb loss research, but often yield contradictory findings across modern ankle-foot devices [19]. While these measures can provide valuable, patient-specific information, such as tracking improvement in function and device preference [31], the results of the present study indicated that performance-based outcome measures were not sensitive to broad differences among ankle-foot device types. However, performance-based outcome measures have shown sensitivity to other prosthetic components, such as prosthetic knee types [37–40], prosthetic sockets [41], and suspension systems [42]. The lack of significance in performance-based outcomes may reflect participants’ ability to maintain performance across ankle-foot device types despite underlying biomechanical variation. According to dynamical systems theory, human movement adapts to environmental and mechanical constraints through flexible coordinative structures. In this context, the change in ankle-foot device type may not have represented a strong enough perturbation to disrupt existing movement strategies, particularly in a relatively high-functioning population. Instead, participants may have adapted their motor patterns to each device to complete the task in a similar manner and within comparable time frames, resulting in non-significant differences. It is plausible that in a lower-functioning population, the same perturbation may be more disruptive, potentially revealing device-specific differences in outcomes. Thus, more complex or physically demanding tasks may better elicit meaningful device-specific differences in motor behavior.

Current, widely used performance-based measures may be too general to detect device-specific differences in prosthetic performance. More detailed measures, such as continuous biomechanical outcomes, may be necessary to detect these subtle changes. However, more complex biomechanical parameters may not be readily accessible or translatable to the clinic without correlating them with surrogate performance-based measures. Therefore, the development of new, sensitive, and clinically translatable performance-based outcome measures, grounded in real-world prosthetic performance, is a critical next step for useful clinical adoption. With advances in wearable technology and machine learning, this could enable assessment of movement efficiency and adaptability during real-world tasks [43].

### Patient-reported outcomes

Patient-reported preference plays a critical role in the prescription of prosthetic ankle-foot devices. Satisfaction with a prosthesis is closely linked to mobility, device use, and quality of life [3,44]. In both clinical and research contexts, user preference is frequently reported as one of the primary drivers of prosthetic ankle-foot device selection [45,46].

ESR devices currently represent the gold standard for prosthetic ankle-foot prescriptions in the VA and Department of Defense (DoD) healthcare systems [22,47]. However, an interesting dichotomy emerged in the present findings: overall patient-reported preferences did not consistently align with objective biomechanical performance. While the PWR device demonstrated significantly better outcomes in several ankle-specific biomechanical measures, self-reported measures consistently favored non-powered options. This divergence may reflect several perceived drawbacks of the PWR device, including the need for frequent charging, audible noise during ambulation, and increased weight relative to ESR and ART devices [48]. PEQ subscales related to sounds and frustration rated PWR devices least favorable, while ESR devices achieved the highest satisfaction and utility scores. While ESR devices can only return a portion of the stored energy during terminal stance to mimic the spring-like behavior of the biological ankle-foot complex, their lightweight design, reliability, and minimal maintenance likely contributed to greater satisfaction and perceived performance-based benefit. Users of the PWR device may perceive the increased power generation in late stance; however, they may be less attuned to more subtle biomechanical improvements occurring in other phases of gait. This may lead users to prioritize practical and psychosocial considerations over improved biomechanical performance at the ankle when selecting a preferred device. These findings underscore the need to balance the relationship between patient-reported preferences and measures of function. While objective metrics inform performance, patient-reported perceptions often determine long-term acceptance and use. Shared decision-making models that incorporate both may yield more personalized and sustainable prosthetic outcomes [49].

Use of participants’ previously prescribed ESR devices may have introduced inherent bias toward that device type. In addition, some participants who enrolled in this investigation may have trialed multiple ESR devices as part of their standard of care, given that prescription is not driven by reimbursement rates within VA and DoD. Therefore, the ESR device used in this study may have been their preferred device within the overarching ESR category. However, the approach in this study enhanced ecological validity by reflecting real-world clinical practice and allowed for broader generalization to the ESR category. Future analyses will explore intra-category variation among ESR devices to determine whether user familiarity or design differences drive these preferences.

### Biomechanical outcomes

Given the wide range of potential biomechanical parameters available for analysis [50], this study focused on variables with broad use in lower limb loss research and high clinical translatability [28]. Of 49 biomechanical parameters analyzed, 19 were highly sensitive to device type, with the majority at the ankle. The PWR device demonstrated values closest to controls across many discrete biomechanical parameters, particularly those related to ankle function, consistent with prior studies showing its capacity to replicate the positive work phases of biological ankle motion through active power generation [28,51]. LDA effectively reduced the dimensions to identify key discriminant gait parameters that strongly differentiated device types. Notably, the PWR device was largely separated from the ART and ESR devices in LD1, but there was more variation within the PWR device coefficients compared with the ART and ESR devices. This increased variation may reflect inconsistencies in PWR device tuning across the cohort, leading to differences in peak propulsion timing and reduced coordination between residual limb motion and prosthetic control during push-off [52].

These findings have valuable implications for clinical practice as clinicians can target these discriminant parameters when trialing different devices to identify those that optimize these factors and create a more accurate definition of clinical hallmarks. Subsequently, clinicians can use these discriminant parameters to better define guidelines for clinical management of this population. Moreover, adequate clinical predictors can help clinicians intervene earlier in the prescription process to ensure individuals with lower limb loss reach their highest levels of function in critical parameters. In the long term, these discriminant parameters could also enhance predictive modeling to guide patient-specific prosthetic recommendations.

Notably, hip-level parameters demonstrated the fewest biomechanical parameters sensitive to device type. Hip joint compensations are well documented with the use of prosthetic ankle-foot devices [53,54]. While different ankle-foot devices have unique features that can have direct, quantifiable, and distinct biomechanical improvements in distal joints (e.g., increased ankle power, greater ankle range of motion), these devices do not cross the knee joint, which may decrease their effects on reducing compensations at more proximal joints. Despite unique design features, ESR, ART, and PWR devices are functionally uniarticular and more mechanically similar to the uniarticular soleus (which does not cross the knee joint) than to the biarticular gastrocnemius. This design limitation reduces their ability to fully replicate the dynamic, contractile tissues of the gastroc-soleus complex and likely impedes their ability to produce distinctive improvements in hip biomechanics to reduce asymmetries and compensations [55,56]. Future biomimetic ankle-foot designs [57,58] that reproduce multi-joint muscle behavior may provide broader biomechanical benefits.

### Future directions

While this study provided a high-level analysis of key differences across ankle-foot device categories, ongoing work will focus on intra-category comparisons, particularly among ESR devices, which represent the most frequently prescribed and diverse group. Identifying parameters that are sensitive to devices within a single category could further enhance and refine clinical prescription precision. Future analyses will also include more complex biomechanical parameters, such as measures of continuous coordination and stability, intact limb loading [59], and roll-over shape. To facilitate clinical translation, advanced biomechanical parameters shown to be sensitive to device type should be correlated with surrogate physical measures that are easier to implement in routine clinical practice. Additionally, prediction modeling analyses aimed at identifying the strongest independent predictors of optimal prosthetic prescription outcomes will be addressed in future work as part of the broader dissemination plan for this study.

Although identifying distinct parameters can help guide prescription, implementation in practice remains hindered by the challenges of device trialing, including time-consuming hardware adjustments, vendor dependencies, and limited access to diverse device inventories, which collectively restricts the ability to systematically and efficiently determine the optimal ankle-foot device for each user [4]. Prosthetic device emulators may help address these limitations by allowing rapid, software-based trialing of a wide range of emulated candidate devices, significantly reducing time and inventory demands [60,61]. With rapid iteration of emulated devices, these robotic systems provide the ability to generate data-driven, individualized insights, supporting more objective and precise device selection.

## Conclusions

The results of this study have important implications for clinical prescription of ankle-foot prostheses. A preliminary set of distinct biomechanical and patient-reported parameters was identified as sensitive to differences in device type. These clinically meaningful parameters provide a foundation for developing more precise, evidence-based prescription guidelines. Integrating objective performance measures with user-reported outcomes can enable clinicians to more accurately evaluate ankle-foot function and tailor device selection to individual patient needs. Ultimately, aligning patients with the most appropriate prosthetic technology has potential to lower costs, reduce healthcare utilization, and improve both physical and psychosocial outcomes for individuals with transtibial limb loss.

## Limitations

This study included a convenience sample of experienced prosthesis users who were generally high functioning and in stable health. As such, the findings may not be fully generalizable to individuals with lower limb loss who are lower functioning, newly amputated, or undergoing active physical rehabilitation. However, the enrolled cohort reflects a population commonly treated in routine limb loss care and most likely to seek new prosthetic prescriptions. Socket suspension was not standardized across participants, which could influence performance. However, to mitigate this factor, suspension was held constant across duplicated sockets within each participant. Although data processors were blinded to device type, participants and clinicians were not, given the distinct mechanical and aesthetic characteristics of the ankle-foot devices, which may have introduced bias into patient-reported outcomes. All participants underwent a standardized one-week acclimation period with each device prior to evaluation. Because participants used their previously prescribed or current ESR device, their longer cumulative experience with that device type could have influenced performance and perception. The sufficiency of a one-week acclimation period also remains uncertain; a recent systematic review on adaptation for new lower limb prosthetic devices found no consensus regarding optimal duration [62], highlighting the need for additional research to establish evidence-based acclimation standards. Despite these limitations, the multicenter design and large sample of prosthesis users provide one of the most comprehensive evaluations of prosthetic ankle-foot device performance across functional, patient-reported, and biomechanical domains.

## Supporting information

S1 Protocol Initial institutional review board-approved protocol. (PDF)

S1 Table Performance-based outcomes for each ankle-foot device type. Linear mixed model estimates and paired comparisons are shown for the 6-minute walk test (6MWT), Amputee Mobility Predictor (AmpPro), Timed Up and Go (TUG), Four Square Step Test (4SST), Stair Assessment Index (SAI), and Hill Assessment Index (HAI) measures.
(DOCX)

S2 Table Patient-reported outcomes for each ankle-foot device type. Linear mixed model estimates (SE) and paired comparisons are shown for the 12-Item Short Form Health Survey (SF-12), Prosthesis Evaluation Questionnaire (PEQ), and Orthotics and Prosthetics Users’ Survey (OPUS) measures.
(DOCX)

S3 Table Demographics of the biomechanical subset. Participant characteristics are shown as mean (SD) for continuous variables and n (%) for categorical variables.
(DOCX)

S4 Table Biomechanical outcomes across ankle-foot devices at each speed. Linear mixed model estimates (SE) and paired comparisons are shown for the 19 unique biomechanical parameters at the ankle, knee, and hip across the three walking speeds for the subset of 29 participants. Percent change from control indicates differences relative to control values. The final column identifies which device had the lowest percent change from control for each parameter. Units are included where relevant.
(DOCX)

S1 File CONSORT 2025 reporting checklist. (DOCX)
