# Supplementary material for: Toward evidence-based prescription of prosthetic ankle-foot devices: A multisite randomized crossover trial identifying performance-based, patient-reported, and biomechanical parameters sensitive to device type
Source: PLoS One. 2026 Jul 2;21(7):e0352644. doi: 10.1371/journal.pone.0352644 (PMC13327263; doi:10.1371/journal.pone.0352644)
Supplement: S1 Protocol — (PDF) [file pone.0352644.s001.pdf]

**Background**

As of June 2015, there have been 1,667 Service Members with combat related amputations treated at the three major United States Military Treatment Centers since the start of Operation Iraqi Freedom (OIF), and into Operation Enduring Freedom (OEF) and Operation New Dawn (OND) (1). More than half of these Service Members are now Veterans, and 95% of OEF/OIF/OND Veterans with limb loss use the VA Healthcare System within 5 years of separation from active duty (2). VA now has a responsibility to serve this unique population for their lifetime, as well as provide care to an additional 40,000 Veterans that have lower extremity limb loss at levels proximal to the ankle (3). For those receiving prosthetic foot-ankle devices, the providers within the amputation care team have well over 100 choices of commercially available prosthetic feet to prescribe (4). In the constantly evolving climate of prosthetic devices, there are limited guidelines available to aid clinicians in prescribing the appropriate components, which is a critical unmet need in the field of prosthetics in the VA and military healthcare systems (5).

The standard of care of VA and DoD amputation care teams, supported by the available literature and clinical consensus, is to prescribe Energy Storing and Returning (ESR) feet (Figure 1) to Veterans and Service Members regardless of guidelines established by Centers for Medicare & Medicaid Services (CMS). This means that ESR ankle-foot units, which kinetically and kinematically out perform traditional options such as the Solid Ankle Cushion Heel (SACH) and Stationary Attachment Flexible Endoskeletal (SAFE) feet (6), are recommended and/or prescribed to all Veterans and Service Members that are ambulatory. This broad clinical decision making is supported by Nielson et al, whose findings on the first ESR ankle-foot unit, the “Flex-Foot”, demonstrated significant improvement in walking speed and a reduction in the energy cost of ambulation over traditional prosthetic foot options (6). ESR feet have also been shown to improve stair ascent initiation by reducing the time of initial double-limb support (7), reducing the requisite activation for propulsory muscles (8) ,and, as the name would indicate, improving elastic response (9). VA and Military healthcare systems affiliated with this proposal have adopted this current standard of clinical practice, but concur there is insufficient evidence to support prescription of specific prosthetic feet within the overarching classification of ESR ankle-foot devices (10-12).

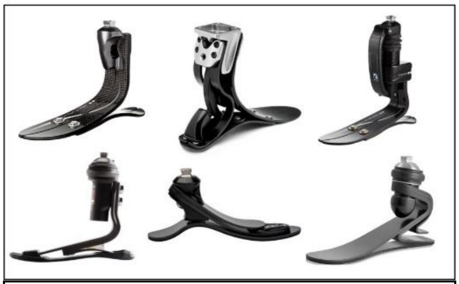

Figure 1. Common examples of the over 100 commercially available ESR feet

There are less commonly prescribed, more advanced ESR options commercially available, including articulating ankles that can present as a passive hydraulic, microprocessor-controlled, or powered units (Figure 2). Articulating ankles in prosthetic feet have been traditionally thought to decrease energy return for individuals with transtibial amputation (TTA), but recent evidence on current articulating ESR ankle-foot units (i.e. Endolite Echelon, Fillauer Motion Foot, Freedom Innovations Kinterra, Otto Bock Triton Smart Ankle, Ossur Proflex) and microprocessor controlled ESR ankle-foot units (i.e. Endolite Elan, Ossur Propriofoot, Hosmer Raize), suggest that articulating hydraulic and microprocessor-controlled non-powered ankle-foot units can improve ambulation on level ground as well as on inclines and stairs (10). De Asha et al. described improved progression of the center of pressure in a prosthetic foot with a hydraulic ankle unit in a population of individuals with TTA and transfermoral amputation (TFA) (13), and Portnoy et al. found a decreased load at heel strike when using and ESR hydraulic ankle-foot unit (14). Fradet et al. reported that a microprocessor controlled ankle improved kinematics in ramp ascent and made patients feel safer during ramp descent (15). This evidence suggests that Veterans and Service Members with TTA that negotiate specific environments may benefit from an articulating ankle ESR foot over a fixed-ankle ESR foot.

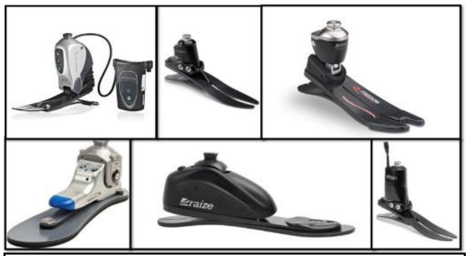

Figure 2. Articulating ankle ESR feet, clockwise from top left Propriofoot, Echelon, Kinterra,

Commercially available ESR feet with active (i.e. powered) plantarflexion (i.e. iWalk BiOM) have potential biomechanical benefits to the user beyond that of the ESR or articulating ankle ESR options (Figure 3). The iWalk BiOM (Bedford, MA) works to replicate the dynamic contractile tissues of the gastroc-soleus complex for individuals with a lower extremity amputation (16). This biomimetic prosthetic device has the ability to normalize ankle power, potentially reducing kinetic asymmetries that lead to musculoskeletal

imbalances, thus improving physical function (16). Grabowski and D'Andrea noted that individuals with TTA using the BiOM had a reduction in peak resultant force and knee adduction moment on the unaffected leg during level-ground walking, potentially limiting the risk of secondary musculoskeletal comorbidities which are common in this population (11, 17-20).

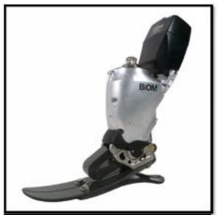

Figure 3. ESR foot with powered plantarflexion

Despite the noted advances in prosthetic technology, much of the research efforts and clinical practice have focused on the design and function of prosthetic technology, rather than understanding which devices are most appropriate to prescribe for individuals with lower limb amputation. Prescription of specific ankle-foot units within VA and DoD healthcare systems, as well as the private sector, is often based on anecdotal evidence or manufacturer driven research (21-23). Furthermore, limitations in the research studies conducted to date, including small sample sizes and non-standardization of feet, make it difficult to directly apply scientific evidence to clinical decision making. This proposal will fill this critical unmet need. Through a multi-centered clinical trial (n =120), the standardization of prosthetic foot characteristics, and matching real-world testing environments, we believe we will be able to come to statistically significant results  to determine which patient goals and outcome measures are most indicative in yielding the most successful and appropriate ankle-foot prosthesis prescription. As such, this study will use step activity monitors, a battery of physical and subjective functional outcome measures, along with biomechanical kinetic and kinematic data to facilitate evidenced-based practice that will be used for a lifetime of care delivery to our Veterans and Service Members living with TTA.  This will be addressed through a four center investigation capturing 120 participants with TTA, which will be largest study of this kind ever conducted. The distribution of sites include VA and DoD covering various geographical and demographical locations.

**Preliminary Data: A Controlled, Longitudinal Evaluation of the iWalk BiOM (PI: Jason Maikos, PhD)**

VA New York Harbor Healthcare System (VANYHHS) is currently conducting a 12-month longitudinal investigation of the BiOM in individuals living with TTA. The primary objective of the investigation is to clinically test and quantitatively evaluate the biomechanical efficacy, functional performance, and daily use of individuals with TTA utilizing the BiOM for 12-months through kinematic and kinetic analysis, functional measures, and step activity monitoring, respectively, many of the same measures that will be collected for this proposal. Figure 4 shows that after 6 months of BiOM use, kinetic asymmetries of the sagittal plane hip kinetics at day 30 have resolved (Figure 3 C&F). Both the sagittal plane hip moment and power have become symmetrical to the intact side, suggesting that the increased power of the prosthetic ankle has reduced the need for hip compensatory strategies.

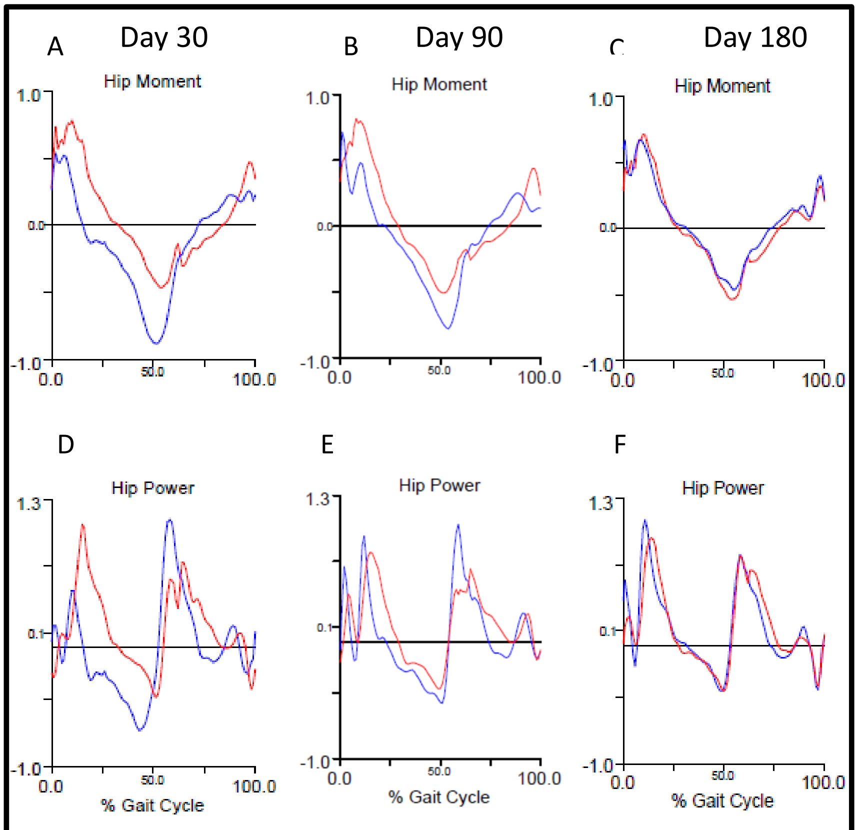


Figure 4: *Sagittal plane hip plane moments (A-C) and powers (D-F) for a representative subject at days 30, 90 and 180. Red lines represent the means for the BiOM limb. Blue lines represent the means for the intact limb.*

Functional measures also generally improved while utilizing the BiOM. Figure 5 details the results of the 6-minute walk test (6-min walk) (Fig 5A), the four square step test (4SST) (Fig 5B), and the Timed-Up-and-Go (TUG) (Fig 4C) for all enrolled subjects at baseline (ESR foot), as well as after 30 and 180 days of BiOM use. Results show improvements in the 6-min walk distance and speed using the BiOM compared to the ESR foot, while average TUG times did not change

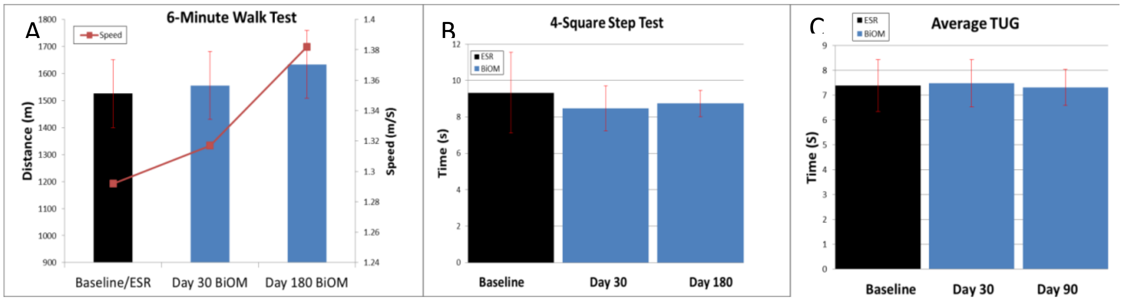


Figure 5: *6-min walk distance and speed (A), Averaged 4SST (B), and average TUG (C) at baseline (ESR foot – black), and after 30 and 180 days of BiOM use (Blue).*

from the baseline condition. The BiOM does not provide an assist during lateral or backward movement, and is heavier than a non-articulating ESR foot. As such, it was expected BiOM use to produce slower 4SST times. However, average 4SST times have exhibited a decrease in 4SST time while utilizing the BiOM. Anecdotally we have also found an approximate 50% user rejection rate of the BiOM despite the functional and biomechanical improvements measured, suggesting that prosthetic prescription guidelines for the BiOM may need to be further refined to determine which patients may not only benefit from the technology, but also accept it.

**Objectives / Specific Aims / Hypotheses**

The purpose of this study is to develop criteria for prosthetic foot prescription for Veterans and Service Members with transtibial limb loss. The objectives are as follows:

- Determine the appropriate functional outcome tests and measures to support the prescription of a type of ESR (non-articulating, articulating or active plantarflexion) prosthetic ankle-foot for a Veteran or Service Member with transtibial limb loss.
- Correlate patient goals and subjective measures with objective data to determine the appropriate prosthetic ankle-foot category that will facilitate the greatest overall function to the user.
- Develop criteria for the appropriate prescription of non-articulating ESR, articulating ESR, and active plantar flexion ESR ankle-foot units.

**Specific Aim 1 – To evaluate the differences in functional performance and subjective outcomes between non-articulating ESR, articulating ESR, and active plantarflexion ESR feet.**

Hypothesis 1.1 Subjects will demonstrate improvement and preference for articulating ESR and active plantar flexion ESR feet over the non-articulating ESR feet for community mobility, particularly for those that regularly negotiate inclines and declines.

Hypothesis 1.2 Active plantar-flexion ESR feet will result in better performance metrics for mobility based outcome measures.

Hypothesis 1.3 Subjects will have decreased low back and proximal joint pain scores as determined by the visual analog scale within the Prosthetic Evaluation Questionnaire (PEQ) when using the active plantarflexion ESR foot.

Hypothesis 1.4 There will be no significant differences in functional outcome measures within all tested non-articulating ESR feet regardless of specific foot assigned.

Hypothesis 1.5 There will be no significant differences in functional outcomes within all articulating ESR feet tested regardless of specific foot assigned.

**Specific Aim 2– To evaluate the kinematic and kinetic differences of non-articulating ESR, articulating ESR, and active plantar flexion ESR feet in individuals with transtibial amputation**

Hypothesis 2.1 Plantarflexion forces (i.e. ground reaction forces) at push off /terminal stance will be most symmetrical to the intact limb for active plantarflexion devices, followed by non-articulating ESR feet, and lastly articulating ESR devices.

Hypothesis 2.2 Biomechanical symmetry in gait while negotiating a decline will be greater for both active plantarflexion ESR and articulating ankle ESR conditions compared to non-articulating ESR feet.

Hypothesis 2.3 Biomechanical symmetry in gait while negotiating an incline will be greatest for devices with active plantarflexion, followed by articulating devices, and lastly non-articulating ESR feet.

Hypothesis 2.4 Ambulation at a faster than self-selected walking speed will yield decreased symmetry in articulating ankle ESR and non-articulating ESR feet but not with the active plantar flexion ESR feet.

Hypothesis 2.5 Articulating ESR feet will not yield significant differences in gait symmetry regardless of specific foot assigned within the articulating ESR category.

**Specific Aim 3 – To correlate biomechanical findings with functional and subjective outcome measures and with user preference determined through activity monitoring and user satisfaction to develop guidelines for appropriate prosthetic prescription.**

Hypothesis 3.1 Individuals that commonly negotiate ramps, hills, inclines or declines will show preference for an articulating ankle ESR and/or active plantarflexion ESR feet over non-articulating ESR feet

Hypothesis 3.2 Individuals that have higher step counts, but do so at a low activity level will prefer the active plantarflexion ESR ankle-foot prosthesis.

Hypothesis 3.3 The majority of subjects will chose to utilize a single prosthesis for all activities, and not use different prostheses for different activities, as measured through step activity monitoring and daily activity logs.

Hypothesis 3.4 Step activity performance will, and age will not, correlate with prosthetic feet category that best optimizes function.

The end point of this study is to generate findings that offer new knowledge that can be developed into clinical practice guidelines, and/or new prescription algorithms for prosthetic ankle-foot units. This information will lead to clinical prescriptions that facilitate achievement of maximal functional ability. Plainly stated, criteria will be created to assist the amputation care team in prescribing appropriate advanced prosthetic ankle-foot systems based on specific patient needs, specific patient goals, in additional to the individual current physical abilities.

**Research Design:**

This investigation will be a prospective multi-center study**,** including the VANYHHS, VA Puget Sound Healthcare System (Seattle VA), James A. Haley VA Medical Center (Tampa VA), and Walter Reed National Military Medical Center (WRNMMC). All sites currently have access and have conducted research on the targeted population. Recruitment of subjects will be done through IRB approved methods.

**Inclusion Criteria:**

- Veteran or Service Member with unilateral TTA
- Subject uses a prosthesis with a well-fitting socket for a minimum of one month
- Each subject must be able to achieve a "No Helper" score on the Functional Independence Measure (FIM) for the 3 mobility items (locomotion, transfers, stairs)

**Functional Independence Measure for Inclusion**:

All subjects must be able to achieve a minimum score of a "modified independence" for the 3 mobility items.
Locomotion: Must be for minimum walking score of "modified independence". A subject may use a wheelchair part time, but FIM inclusion criteria for locomotion must be specifically for "walking" or "both".

**Exclusion criteria**:

- subjects with active wounds/ulcers or significant musculoskeletal comorbidities on their intact limb that would impair their ability to participate in all functional outcome measures
- subjects with knee flexion contracture > 5 degrees
- any comorbidity that results in rapid limb volume changes (i.e. end stage renal disease with dialysis),
- a poorly fitting socket
- cognitive deficit(s) or mental health pathology limiting a subject's ability to participate fully in the study
- subjects who are unable or unwilling to comply with all research visits
- women who are pregnant or who plan to become pregnant in the near future
- subject weighs more than product weight load maximums
- score of less than "modified independence" on any of the FIM mobility items

**Foot Selection:**

Prosthetic ankle-foot units included in this study will be grouped in to three categories: 1) Non-articulating ESR - this group will be any qualifying ESR ankle-foot unit that is commercially available. This will typically be the foot the subject is currently using. This group contains over 100 commercially available prosthetic feet, and will be randomly chosen from all available options that fit the subject's anatomy if the user does not current have a non-articulating ESR foot. 2) Articulating ESR – this group includes all commercially available options

that have an articulating ankle and also have ESR qualities, currently Endolite Echelon, Fillauer Motion Foot, Freedom Innovations Kinterra, Endolite Elan, Ossur Propriofoot, Hosmer Raize, Otto Bock Triton Smart Ankle, and Ossur Proflex (Figure 2) meet this criteria. 3) Active (Powered) Plantarflexion - the third group will contain all commercially available ESR ankle-foot units with active plantarflexion. Currently, the iWalk BiOM is the only foot that would be classified into the Active Plantarflexion ESR group. If new prosthetic feet-ankle units become commercially available during the course of this study, they will be added to the appropriate groups.

**Justification for inclusion of all commercially available feet**

*Non-articulating ESR Group:* Many non-articulating ESR prosthetic feet have different mechanical features (i.e. torsion, vertical shock) that provide for specific HCPCS code classification as explained in AOPA's "Prosthetic Foot Project" (24). However, to the best of our knowledge, while there may be subtle mechanical differences between these feet, no studies to date have shown any significant differences in impact on function between different non-articulating ESR feet. More specifically, while manufacturer marketing and patient subjective feedback can often report differences between prosthetic feet, there are no published differences in function during level ground ambulation, stair negotiation, or incline/decline ambulation using non-articulating ESR feet (10-12). Clinical consensus is that patients do have individual preferences for specific non-articulating ESR ankle-foot units, but there is no current metric(s) to predict this. To limit the study to one, or just a few non-articulating ESR feet, would limit the findings to just that one or a few specific feet chosen to represent a large group when drawing a conclusion. Additionally, most patients seen at the sites included in this study have the ability to trial many different prosthetic feet, as prescription is not driven by reimbursement rates within VA or DoD, and thus the foot the subject enrolls with may be a foot of preference within the larger non-articulating ESR category. Prosthetic feet frequently change, update, and can even be taken off the market for another generation ankle-foot unit. Limiting the foot choices, would also run the risk of not investigating the current trends of what is being prescribed in the amputation care clinics at the four VA and DoD sites in this study.

*Articulating ESR Group*: The articulating ESR group contains mechanical and microprocessor controlled prosthetic ankle-foot units. While there is research comparing these devices to SACH feet and non-articulating ESR feet, there is no current research suggesting any differences between the feet within this group in their effect on functional performance. These prosthetic ankle-foot units have articulation ranges from 9 to 50 degrees within this group, and it has been reported that average range of motion in a healthy individual with an intact lower limb is 20-40 degrees for ankle plantar/dorsiflexion (25). There is no published research to-date as to the ideal range of motion for an individual with transtibial limb loss, so all ankle-foot units will be included. Internal analysis within all groups will be conducted at the completion of this study to substantiate or rebut these groupings, and to determine if there are in fact outliers that should be classified differently clinically or in future research.

*Active Plantarflexion ESR Group:* Criteria for this group includes the following

- Commercially available
- Must be articulating
- Provides plantarflexion energy during terminal stance at a rate that is greater than the total energy absorbed (i.e. >100%)

**Research Protocol:**

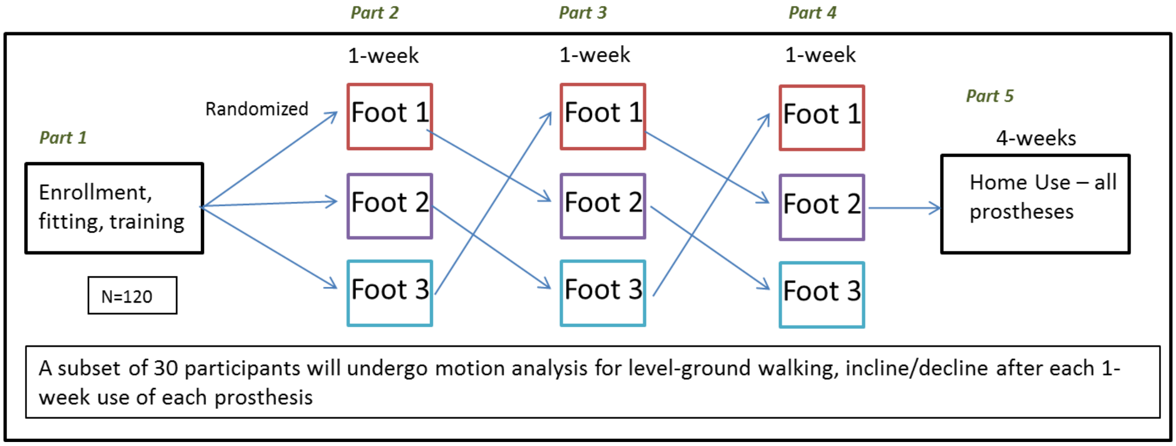


Figure 6 – Project timeline for each subject.

**Part 1a: Screening, Consent, Enrollment, Prosthetic Socket Assessment**

During the first visit, subjects will be screened according to the inclusion/exclusion criteria, consented and enrolled. Demographic information will be collected including, age, height, weight, injury etiology, and time as a prosthesis user. Fit and comfort of each subject's existing socket will be evaluated by the study prosthetist on-site through standardized prosthetic guidelines. Once fit and comfort are confirmed, the study prosthetist will create a mold using plaster of paris or alginate for fabrication of two identical prosthetic sockets.

Between visits 1 and 2, the molds of the existing socket shape and volume will be used to create laminated, definitive sockets, for the two additional prostheses. These sockets will be centrally fabricated at VANYHHS. The same materials as used in the subject's current socket will be used to create each additional socket to eliminate any variance in perceived weight or socket flexibility. Each additional socket will be aligned/programmed with a designated prosthetic foot to manufacturer's specifications by the study prosthetist.

**Randomization of Foot Selection and Order of Evaluation**

After enrollment, the prosthetic devices for each subject, as well as the order in which they will be evaluated, will be randomized using a computer generated algorithm that randomly selects from all available devices and then assigns the order. As determined by subject's weight, height and activity level (activity level rating is based on parameters set by the particular manufacturer/foot), each randomly selected foot will be purchased from the manufacturer by the study coordinator, under the supervision of the principal investigator, at the VANYHHS.

**Part 1b: Static fitting, dynamic fitting and prosthetic alignment**

Subjects will return for fitting of the definitive sockets/prostheses by the study prosthetist. The additional prostheses will initially be bench aligned, based on manufacturer's specifications, for a static fitting on the subject. After successful static fitting is achieved, the dynamic alignment of each prosthesis will occur. The subject will ambulate using each of the duplicate prostheses and adjustments will be made to the alignment, as needed, to achieve an optimal gait pattern, as determined by each subject, site prosthetist and/or research team. Dynamic fitting may take multiple visits, and subjects will progress to the next part of the protocol after an appropriate dynamic alignment has been established.

*Device Specific Training to Meet a Minimum Standard of Performance*

After appropriate fit and alignment of each prosthetic device has been established, all subjects will undergo device specific training by a qualified clinician using each foot to meet a minimum standard of functional performance. The minimum standard will be defined using the mobility section of the FIM with each prostheses. The participant must score a minimum of "modified independence" to advance in the study (detailed in the Intervention Plan). If the participant fails to achieve this outcome, gait training/rehabilitation to enhance the participant's use of their prosthetic foot will be provided until they are able to meet the minimum standard of performance (further outlined in the Intervention Plan). The process of device specific training may take multiple visits. Part 2 of the protocol will begin when a subject meets the minimum standard of performance.

**Specific Aim 1**: Assessment of function using ESR, Articulating ESR and Active Plantarflexion ESR ankle-foot prostheses

Parts 2-4 of the study will address Specific Aim 1. Part 2 will begin with issuing the first of the 3 well-fitting prostheses according to the randomization schedule, including a 1 week acclimation period. At the conclusion of this acclimation period, a battery of outcome measures and surveys (outlined below) will be collected. For Part 3, the subject will receive the second prosthesis according to the established randomization schedule and the initial prosthesis will be returned to the study prosthetist. The subject will again have a one week acclimation period followed by the collection of the same battery of outcome measures and surveys. The third prosthesis will be issued during part 4, and the second prosthesis will be returned to the study prosthesis. The subject with receive the same one week acclimation followed by the battery of outcome measures and surveys.

**Outcome Measures**

The battery of functional outcome measures and surveys/guided interviews delivered after each acclimation period is delivered by a local researcher. Functional outcome measures include the 6-Min Walk, TUG, 4SST,

Amputee Mobility Predictor (AmpPRO), stair assessment index (SAI), and hill assessment index (HAI). Surveys/guided interview include Prosthetic Evaluation Questionnaire (PEQ), Short Form-12 (SF-12), and Orthotics and Prosthetics User Survey (OPUS).

- *6-Min Walk:* This test measures the distance an individual can walk in 6 minutes without help or encouragement. The 6-minute walk test is valid and reliable metric. It correlates with physical function and functional capacity (26, 27). This test has been proven to have good inter-rater and intra-rater reliability in transtibial amputees (26).
- *TUG*: The TUG measures the time taken by an individual to stand up from a standard arm chair, walk a distance of 10 feet, turn, walk back to the chair, and sit down. The TUG is both reliable and valid in a population with unilateral transtibial and transfemoral amputation (28). Additionally, the TUG has also been used as a measure to determine risk of falling (29, 30). This measure has also shown to correlate well with other measures of physical functioning (31).
- *4SST*: The 4SST is a higher order complex task assessing dynamic balance (32, 33).
- AmpPRO: The AmpPRO is a 21-item instrument designed to measure basic prosthetic mobility of individuals with lower extremity amputation (34).
- HAI: The HAI is a rated qualitative scale on how an individual with lower limb amputation negotiates up and down an incline (35).
- SAI: The SAI is a rated qualitative scale for stair negotiation (35).
- PEQ: The PEQ is self-report visual analog scale-style questionnaire for persons with lower limb amputations who use a prosthesis. It is used to evaluate the prosthesis and life with the prosthesis. It consists of 82 items with nine functional domain scales including ambulation, residual limb health, utility, appearance, sounds, frustration, perceived response, social burden and well-being. The PEQ also contains items beyond the subscales including satisfaction, pain, transfers, prosthetic care, self-efficacy, and importance (36).
    - PEQ-Addendum: This is an addendum to the PEQ, and it is a self-reporting measure to assess any stumbles and/or falls that occur during study protocol participation.
- SF-12: The SF-12 can be used to measure the relationship between physical and mental health functioning and the social determinants of health (37).
- OPUS: OPUS is a set of self-report instruments that assess functional status, quality of life, and satisfaction with devices and services that can be used in an orthotics and prosthetics clinic. It can be used with patients with varying levels of lower limb function, quality of life, and satisfaction with devices and services (38).

**Research Monitoring**

The Research Monitor, Roxanne Disla, OT, at the VA NY Harbor Healthcare System is responsible for overseeing the safety of the research and reporting findings to the IRB. The Research Monitor will review all unanticipated problems involving risks to subjects or others associated with the protocol and provide an independent report of the event to the IRB. Reports of the progress of the study will be completed by the Research Monitor and submitted to the IRB and HRPO (Human Research Protection Office at the DoD), as needed. This includes an analysis of any stumbles and/or falls that occur with any of the enrolled research subjects throughout their participation in the protocol activities. The Research Monitor will discuss the research protocol with the investigators; shall have authority to stop the research protocol in progress, remove individual human subjects from a research protocol, and take whatever steps are necessary to protect the safety and well-being of human subjects until the IRB can assess the monitor's report; and shall have the responsibility to promptly report their observations and findings to the IRB official and the HRPO.

**SA1: Data and Statistical Analysis**

*Hypothesis 1.1 Subjects will prefer articulating ESR and active plantar flexion ESR ankle-foot units over the non-articulating ESR option for community mobility, particularly for those that regularly negotiate inclines and declines.* Preference will be demonstrated in the PEQ mobility questions. Across the study population, outcomes will be assessed with descriptive statistics (i.e. means and standard errors) and compared between

each intervention prosthesis. Inferential statistics for ordinal data (SAI, HAI) will be conducted with a repeated measures Friedman test (α=.05) and a Dunn post hoc test at a 95% confidence interval. To analyze the bivariate relationships between the factors of interest and the outcome measures (SAI, HAI), t-tests will be performed.

*Hypothesis 1.2 Active plantar-flexion ESR feet will result in better performance metrics for mobility based outcome measures.* For each functional outcome measures (6-min walk, TUG, AmpPRO, SAI and HAI) we will perform a repeated-measures analysis of variance (ANOVA) to compare the 3 testing conditions. When the ANOVA reveals significant differences ($p < 0.05$), we will apply pairwise comparisons using related pairs t-tests to identify the source of differences ($p < 0.05$). We will compute the difference between a given pair of sessions for the subject groups and determine the mean difference for subject groups.

*Hypothesis 1.3 Subjects will have decreased pain scores as determined by the visual analog scale (VAS) within the Prosthetic Evaluation Questionnaire when using the active plantarflexion ESR ankle-foot prosthesis*
The PEQ contains a VAS for pain within the evaluation. Spearman correlations, a statistical measure of the strength of a monotonic relationship between paired data will be calculated to correlate data between pain and functional outcome values across the 3 groups. Linear mixed-effects models will be used to identify statistically significant differences in pain scores and functional outcome variables. In the case of significance, pair-wise comparisons will be tested for significance using linear contrasts with Tukey's Honestly Significant Difference or applying Bonferroni's correction. Finally, stepwise multiple regression will be performed with VAS pain as the dependent variable The independent variables will be comprised of functional parameters and condition (foot type). Significance will be set at $p<0.05$. Variables will be eliminated if p is > 0.1.

*Hypothesis 1.4 There will be no significant differences in functional outcome measures within all tested non-articulating ESR feet regardless of specific foot assigned.*

*Hypothesis 1.5 There will be no significant differences in functional outcomes within all articulating ESR feet tested regardless of specific foot assigned.*
Functional measures will be analyzed by multilevel (site) repeated measures analysis of variance. Because order of prosthesis will be randomized, there should be no order effect. However, order will be included in the initial analysis to test this assumption. When the ANOVA reveals significant differences ($p < 0.05$), we will apply pairwise comparisons using related pairs t-tests to identify the source of differences ($p < 0.05$).

**Specific Aim 2:**
Specific Aim 2 will be addressed through a subset of n=30 subjects which will undergo kinematic and kinetic biomechanical evaluations at the completion of each acclimation period for Parts 2-4. This will include kinetic and kinematic evaluation on level ground walking, and additional kinematic evaluation on inclines and declines.
**Gait Analysis**
***Instrumentation:*** Gait analysis will be performed at the VISN 3 Gait and Motion Analysis Laboratory at VA NYHHCS and the Biomechanics Laboratory at WRNMMC. The VISN 3 Gait and Motion Analysis Laboratory is a 1,440 square foot space comprised of an eight-camera infrared motion analysis system (Vicon Inc. Oxford, UK) with 4 multi-axis force plates (2 AMTI – Waterford, MA and 2 Kistler - Winterthur, Switzerland). The system records the position of passive reflective markers at a rate of 120 Hz, and force plates sample data at a rate of 3,000 Hz. At WRNMMC, the Amputee Care Service within the Department of Rehabilitation houses the Center for Performance and Clinical Research (CPCR) which includes the Biomechanics Laboratory. The Biomechanics Laboratory is a 1,200 square foot space comprised of a 27 camera motion capture system (Vicon Inc. Oxford, UK), and 6 6-degree-of-freedom force platforms (AMTI, Waterford, MA). Visual3D software (C-Motion Inc, Rockville, MD) will be utilized for analysis of 3D motion capture data. It is hardware independent and provides clinically validated, consistent results.
*Kinematic and Kinetic Data Capture:* Gait analysis will be performed after each acclimation period completing Part 2, Part 3 and Part 4 of the study. Wearing shorts, each test subject be provided with biomechanically neutral shoes that offer neither supinatory nor pronatory control. A custom, full-body passive reflective marker set will be placed on each subject, which tracks each segment independently, allowing for the accurate

measurement of movements. A total of 60 markers will be placed or digitized on the trunk and extremities. Marker placements for the prosthetic limb will be matched to those of the intact leg. The cluster technique will be employed to minimize the surface to bone displacements for the thigh, shank, and upper arm mounted markers (39). As such, tracking clusters will be placed bilaterally on the thigh, shank (tibial crest), and upper arm. Functional joints will also be calculated for the intact ankle, knee, and hips, adapted from Schwartz and Rozumalski (40).

***Procedure:*** During each experimental session, subjects will walk at 3 speeds across a 10 meter instrumented walkway until 5 trials when each foot makes full contact with each force plate for at least two sequential foot strikes. Since kinetic outcome measures are speed dependent, subjects will ambulate with controlled speeds determined by normalizing to each subject's intact limb length (41). Subjects will walk at their defined median Froude speed (FR3) and at one speed below and one speed above the median (FR2 & FR4), respectively. Auditory feedback will be provided to the subject in the form of a tone to ensure that they are walking at the correct speed. Subjects will be asked to repeat the walking trials until 5 acceptable trials for each speed are collected. The main purpose of this session is to collect joint motion data, force, torque, and power on subjects during each test condition. Segment ranges of motion, velocities and accelerations, and corresponding hip, knee, and ankle joint moments of force and generated/absorbed powers will be computed using inverse dynamics methods. Averages, maximum and minimum values, standard deviations and coefficients of variation will be computed for the values attained per gait cycle over each trial and for each set of trials. Spatial and temporal parameters, such as stride and step lengths, step times and velocities will also be recorded.

Additionally subjects in this subset will perform ramp ascent and descent under the collection of kinematic data. This will be performed on a standardize ramp of 1:6 rise:run ratio, which is steeper than ADA guidelines. Subjects will be instructed to ambulate at a comfortable self-selected walking speed. This will include 5 trials ascending and 5 trials descending.

**SA2 Data Analysis:** The reflective marker positions will be digitized using motion tracking software (Vicon, Oxford, UK). A 15-segment (head, trunk, pelvis, and bilateral upper/lower arm, hand, thigh, shank, and feet) rigid body model will be created based on the skin-mounted markers and functional joints. Local coordinate systems for each segment will be defined using the International Society of Biomechanics (ISB) recommendations (42, 43). The data of 5 acceptable walking trials of each participant at each speed will be processed using Visual3D (C-Motion Inc., Rockville, MD). Marker data will be filtered with a 6 Hz Butterworth low-pass filter. Raw analog data will be filtered using a second-order low-pass Butterworth filter with a 25 hertz cutoff frequency. Visual 3D software will be used to calculate temporal-spatial values, walking velocity, as well as lower extremity kinematics and kinetics. Inverse dynamics analysis is applied to the kinematics of the biomechanical model and to the location, magnitude, and direction of ground reaction forces acting on the foot to calculate lower extremity joint torques and powers, including ankle, knee, and hip power of the biological and prosthetic limbs over stance phase, as well as the frontal plane knee moments for the unaffected legs of all subjects. The resultant ground reaction force will be calculated as the magnitude of the ground reaction force vector.

**SA2 Statistical Analysis:**

To address the hypotheses stated for SA2, several parameters, including temporal-spatial variables (step lengths, stance/swing times, cadence, and stride length), peak angles, moments, and powers for the ankles, knees, and hips during each phase of gait, and other biomechanical parameters (external adduction moment rate, impulse external knee adduction moment, peak vertical ground reaction force, peak resultant ground reaction force, etc.) will be measured and statistically compared across all conditions. Peak values are identified as the greatest local maxima during the first half of stance. We will use linear mixed-effects models to identify statistically significant differences in gait temporal-spatial and biomechanical variables. The fixed effects for our study will be the average differences in gait biomechanical and temporal-spatial variables by prosthetic foot type. For example, we will use a linear mixed effects regression to examine the relationship between intact knee peak EAM and prosthetic foot condition. Peak intact knee EAM will be the dependent variable, while foot condition will be the independent variable and subject by foot condition will be modeled as random effects. In the case of significance, pair-wise comparisons will be tested for significance using linear contrasts with Tukey's Honestly

Significant Difference or applying Bonferroni's correction. We will also use linear mixed effects regression to determine the association between prosthetic peak ankle power and foot condition.

**Specific Aim 3**

Part 5: **Step Activity Monitoring**

Specific Aim 3 will be addressed with Part 5 of this protocol. After completion of Part 2-4, subjects will receive all three prosthetic limbs for use for a period of 30 days. All three limbs will be fitted with a Modus Health step activity monitor (SAM), which is a research-grade instrument for long-term assessment of ambulatory activity during day-to-day life. Subjects will also use an activity log to document their daily activities to allow for correlation between prosthesis used and activity. Subjects are free to use each prosthesis as they chose. Subject prosthetic preference will be based upon total activity time for each prosthesis.

**SA3 Data Analysis:**

Daily step counts and level of activity (low, medium, high) will be collected from each SAM attached to each prosthesis, and daily activity logs will be analyzed to determine which devices were used for specific activities (i.e. everyday use, leisure, housework). Together, this will help determine rationale for patient preference of prosthesis. Additionally these findings will be correlated with previous outcome measures from Parts 2-4.

**Development of Prediction Model:**

In order to develop guidelines for prosthetic prescription of ankle-foot devices, the goal of this proposal is to determine which factors and outcomes are most predictive of optimal performance to meet patient goals. To do this, initial analyses will consist of bivariate cross-tabulations, conducted using chi square analyses, based on clinical hypotheses between each potential explanatory variable. Variable selection for the multivariate model will be informed by bivariate findings. Logistic regression will then be performed with patient preference of prosthesis regressed on each of several domains (i.e. functional levels/outcomes, Activity levels, biomechanical parameters, among others). The models will control for multiple variables simultaneously. The C-statistic corresponding to the area under the receiver operating characteristic (ROC) curve will be used to assess overall predictive value. Theory-driven domain specific models (based on bivariate findings) and one parsimonious stepwise logistic regression model combining the domains will be created. This will help determine whether each distinct component predicts optimal prosthetic prescription outcome. Each domain-specific model will be adjusted for and controlled for confounding factors. The individual variables expressing the components of each domain will be entered as fixed predictors. Finally, a stepwise multiple regression will be applied to identify the set of clinical factors that represent the strongest independent predictors. The stepwise regression will be conducted through a series of domain-specific forward-regression models and one backward regression. The variables significant in these forward selection models will be placed in the backward model and will be removed at $P = .10$. The significant variables from the backward regression will be presented as the strongest independent predictive factors and be used to help guide prosthetic prescription.

**Power Analysis:**

| *Site* | *n* | *n for subset* |
|---|---|---|
| VA New York Harbor Healthcare System (NYC) | 30 | 15 |
| Walter Reed National Military Medical Center (D.C.) | 30 | 15 |
| VA Puget Sound Healthcare System (Seattle) | 30 | 0 |
| James J. Peters VA Medical Center (Tampa) | 30 | 0 |
| **TOTAL** | **120** | **30** |

Figure 7. Total Subjects per site to be seen over the course of 3 year period

Specific Aim 1

Functional measures will be analyzed by multilevel (site) repeated measures analysis of variance. Because order of prosthesis will be randomized, there should be no order effect. However, order will be included in the initial analysis to test this assumption. Assuming an alpha error rate of 5% and within-group standard deviation of 5, 120 subjects will provide over 98% power to detect a 3 unit difference between at least two of the ESR groups for each of the functional measures.

Specific Aim 2
Kinematic and kinetic measures will be analyzed by multilevel (site) repeated measures analysis of variance. Because order of prosthesis will be randomized, there should be no order effect. However, order will be included in the initial analysis to test this assumption. Assuming an alpha error rate of 5% and within-group standard deviation of 10, 30 subjects will provide 72% power to detect a 6-unit difference between at least two of the ESR groups for each of the measures.

Specific Aim 3
This analysis will be performed using paired t-test to determine correlations between prosthesis chosen as per SAM data and which functional outcome measures are indicative of that choice. Additionally pair t-test will be performed between kinematic and kinetic data to again correlate with the prosthetic choice of the subject as demonstrated through the SAM data. Finally all SAM data will be evaluated against the activity journal to determine the roll of a specific activity goal and a specific prosthesis.

References Cited

1. Fischer H. A Guide to U.S. Military Casualty Statistics: Operation New Dawn, Operation Iraqi Freedom, and Operation Enduring Freedom. Washington DC: Congressional Research Service, 2014.
2. Healthcare Inspection: Prosthetic Limb Care in VA Facilities. Washington DC: VA Office of Inspector General, 2012.
3. Fischer H. U.S. Military Casualty Statistics: Operation New Dawn, Operation Iraqi Freedom, and Operation Enduring Freddom. 2013.
4. van der Linde H, Hofstad CJ, Geurts CH, Postema K, Geertzen JHB, van Limbeek J. A Systematic Literature Review of the Effect of Different Prosthetic Components on Human Functioniong with Lower-Limb Prosthesis. Journal of Rehabilitation Research and Development. 2004;41(4):555-70.
5. Linde Hvd, Hofstad CJ, Geurts ACH. A systematic literature review of the effect of different prosthetic components on human functioning with a lower-limb prosthesis. Journal of Rehabilitation Research and Development. 2004;41(4):555-70.
6. Nielson D, Shurr J. Comparison of energy cost and gait efficiency during ambulation in below-knee amputees using different prosthetic feet – a preliminary report. Journal of Prosthetics and Orthotics. 1988;1(1):24-31.
7. Torburn L, Schweiger GP, Perry J, Powers CM. Below-knee amputee gait in stair ambulation. A comparison of stride characteristics using five different prosthetic feet. Clin Orthop Relat Res. 1994(303):185-92. PubMed PMID: 8194232.
8. Ventura JD, Klute GK, Neptune RR. The effect of prosthetic ankle energy storage and return properties on muscle activity in below-knee amputee walking. Gait Posture. 2011;33(2):220-6. doi: 10.1016/j.gaitpost.2010.11.009. PubMed PMID: 21145747.
9. Versluys R, Beyl P, Van Damme M, Desomer A, Van Ham R, Lefeber D. Prosthetic feet: state-of-the-art review and the importance of mimicking human ankle-foot biomechanics. Disabil Rehabil Assist Technol. 2009;4(2):65-75. doi: 10.1080/17483100802715092. PubMed PMID: 19253096.
10. Agrawal V, Gailey RS, Gaunaurd IA, O'Toole C, Finnieston AA. Comparison between microprocessor-controlled ankle/foot and conventional prosthetic feet during stair negotiation in people with unilateral transtibial amputation. J Rehabil Res Dev. 2013;50(7):941-50. doi: 10.1682/JRRD.2012.05.0093. PubMed PMID: 24301431.
11. Agrawal V, Gailey R, O'Toole C, Gaunaurd I, Finnieston A. Influence of gait training and prosthetic foot category on external work symmetry during unilateral transtibial amputee gait. Prosthet Orthot Int. 2013;37(5):396-403. doi: 10.1177/0309364612473501. PubMed PMID: 23364890.
12. Hafner BJ, Sanders JE, Czerniecki JM, Fergason J. Transtibial energy-storage-and-return prosthetic devices: a review of energy concepts and a proposed nomenclature. J Rehabil Res Dev. 2002;39(1):1-11. PubMed PMID: 11926321.
13. De Asha AR, Munjal R, Kulkarni J, Buckley JG. Walking speed related joint kinetic alterations in trans-tibial amputees: impact of hydraulic 'ankle' damping. J Neuroeng Rehabil. 2013;10:107. doi: 10.1186/1743-0003-10-107. PubMed PMID: 24134803; PubMed Central PMCID: PMCPMC4015832.
14. Portnoy S, Kristal A, Gefen A, Siev-Ner I. Outdoor dynamic subject-specific evaluation of internal stresses in the residual limb: hydraulic energy-stored prosthetic foot compared to conventional energy-stored prosthetic feet. Gait Posture. 2012;35(1):121-5. doi: 10.1016/j.gaitpost.2011.08.021. PubMed PMID: 21955382.
15. Fradet L, Alimusaj M, Braatz F, Wolf SI. Biomechanical analysis of ramp ambulation of transtibial amputees with an adaptive ankle foot system. Gait Posture. 2010;32(2):191-8. doi: 10.1016/j.gaitpost.2010.04.011. PubMed PMID: 20457526.
16. Au SK, Herr H, Weber J, Martinez-Villalpando EC. Powered ankle-foot prosthesis for the improvement of amputee ambulation. Conf Proc IEEE Eng Med Biol Soc. 2007;2007:3020-6. doi: 10.1109/IEMBS.2007.4352965. PubMed PMID: 18002631.
17. Norvell DC, Czerniecki JM, Reiber GE, Maynard C, Pecoraro JA, Weiss NS. The prevalence of knee pain and symptomatic knee osteoarthritis among veteran traumatic amputees and nonamputees. Arch Phys Med Rehabil. 2005;86(3):487-93. doi: 10.1016/j.apmr.2004.04.034. PubMed PMID: 15759233.

18. Grabowski AM, D'Andrea S. Effects of a powered ankle-foot prosthesis on kinetic loading of the unaffected leg during level-ground walking. J Neuroeng Rehabil. 2013;10:49. doi: 10.1186/1743-0003-10-49. PubMed PMID: 23758860; PubMed Central PMCID: PMCPMC3685554.
19. Kulkarni J, Gaine WJ, Buckley JG, Rankine JJ, Adams J. Chronic low back pain in traumatic lower limb amputees. Clin Rehabil. 2005;19(1):81-6. PubMed PMID: 15704512.
20. Struyf PA, van Heugten CM, Hitters MW, Smeets RJ. The prevalence of osteoarthritis of the intact hip and knee among traumatic leg amputees. Arch Phys Med Rehabil. 2009;90(3):440-6. doi: 10.1016/j.apmr.2008.08.220. PubMed PMID: 19254609.
21. Hofstad C, Linde H, Limbeek J, Postema K. Prescription of prosthetic ankle-foot mechanisms after lower limb amputation. Cochrane Database Syst Rev. 2004(1):CD003978. doi: 10.1002/14651858.CD003978.pub2. PubMed PMID: 14974050.
22. Stark G. Perspectives on How and Why Feet are Prescribed. Journal of Prosthetics and Orthotics. 2005;17(4):S18-S22.
23. Twiste M, Rithalia S. Transverse rotation and longitudinal translation during prosthetic gait--a literature review. J Rehabil Res Dev. 2003;40(1):9-18. PubMed PMID: 15150716.
24. AOPA'S PROSTHETIC FOOT PROJECT: What It Is, What It Is Not, and What Patient Care Facility Providers/Practitioners Need to Know…. American Orthotic and Prosthetic Association, 2010.
25. Perry J. Gait Analysis: Normal and Pathological Function. New Jersey: Slack Incorporated; 1992.
26. Brooks D, Hunter JP, Parsons J, Livsey E, Quirt J, Devlin M. Reliability of the two-minute walk test in individuals with transtibial amputation. Arch Phys Med Rehabil. 2002;83(11):1562-5. PubMed PMID: 12422326.
27. Butland RJ, Pang J, Gross ER, Woodcock AA, Geddes DM. Two-, six-, and 12-minute walking tests in respiratory disease. Br Med J (Clin Res Ed). 1982;284(6329):1607-8. PubMed PMID: 6805625; PubMed Central PMCID: PMCPMC1498516.
28. Schoppen T, Boonstra A, Groothoff JW, de Vries J, Göeken LN, Eisma WH. The Timed "up and go" test: reliability and validity in persons with unilateral lower limb amputation. Arch Phys Med Rehabil. 1999;80(7):825-8. PubMed PMID: 10414769.
29. Whitney JC, Lord SR, Close JC. Streamlining assessment and intervention in a falls clinic using the Timed Up and Go Test and Physiological Profile Assessments. Age Ageing. 2005;34(6):567-71. doi: 10.1093/ageing/afi178. PubMed PMID: 16267180.
30. Kristensen MT, Foss NB, Kehlet H. Timed "up & go" test as a predictor of falls within 6 months after hip fracture surgery. Phys Ther. 2007;87(1):24-30. doi: 10.2522/ptj.20050271. PubMed PMID: 17142643.
31. Podsiadlo D, Richardson S. The timed "Up & Go": a test of basic functional mobility for frail elderly persons. J Am Geriatr Soc. 1991;39(2):142-8. PubMed PMID: 1991946.
32. Dite W, Connor HJ, Curtis HC. Clinical identification of multiple fall risk early after unilateral transtibial amputation. Arch Phys Med Rehabil. 2007;88(1):109-14. doi: 10.1016/j.apmr.2006.10.015. PubMed PMID: 17207685.
33. Lythgo N, Marmaras B, Connor H. Physical function, gait, and dynamic balance of transfemoral amputees using two mechanical passive prosthetic knee devices. Arch Phys Med Rehabil. 2010;91(10):1565-70. doi: 10.1016/j.apmr.2010.07.014. PubMed PMID: 20875515.
34. Gailey RS, Roach KE, Applegate EB, Cho B, Cunniffe B, Licht S, et al. The amputee mobility predictor: an instrument to assess determinants of the lower-limb amputee's ability to ambulate. Arch Phys Med Rehabil. 2002;83(5):613-27. PubMed PMID: 11994800.
35. Hafner BJ, Willingham LL, Buell NC, Allyn KJ, Smith DG. Evaluation of function, performance, and preference as transfemoral amputees transition from mechanical to microprocessor control of the prosthetic knee. Arch Phys Med Rehabil. 2007;88(2):207-17. doi: 10.1016/j.apmr.2006.10.030. PubMed PMID: 17270519.
36. Legro MW, Reiber GD, Smith DG, del Aguila M, Larsen J, Boone D. Prosthesis evaluation questionnaire for persons with lower limb amputations: assessing prosthesis-related quality of life. Arch Phys Med Rehabil. 1998;79(8):931-8. PubMed PMID: 9710165.

37. Burdine JN, Felix MR, Abel AL, Wiltraut CJ, Musselman YJ. The SF-12 as a population health measure: an exploratory examination of potential for application. Health Serv Res. 2000;35(4):885-904. PubMed PMID: 11055454; PubMed Central PMCID: PMCPMC1089158.
38. Heinemann AW, Bode RK, O'Reilly C. Development and measurement properties of the Orthotics and Prosthetics Users' Survey (OPUS): a comprehensive set of clinical outcome instruments. Prosthet Orthot Int. 2003;27(3):191-206. PubMed PMID: 14727700.
39. Lathrop RL, Chaudhari AM, Siston RA. Comparative assessment of bone pose estimation using Point Cluster Technique and OpenSim. J Biomech Eng. 2011;133(11):114503. doi: 10.1115/1.4005409. PubMed PMID: 22168744.
40. Schwartz MH, Rozumalski A. A new method for estimating joint parameters from motion data. J Biomech. 2005;38(1):107-16. doi: 10.1016/j.jbiomech.2004.03.009. PubMed PMID: 15519345.
41. Alexander RM. Optimization and gaits in the locomotion of vertebrates. Physiol Rev. 1989;69(4):1199-227. PubMed PMID: 2678167.
42. Wu G, Siegler S, Allard P, Kirtley C, Leardini A, Rosenbaum D, et al. ISB recommendation on definitions of joint coordinate system of various joints for the reporting of human joint motion--part I: ankle, hip, and spine. International Society of Biomechanics. J Biomech. 2002;35(4):543-8. PubMed PMID: 11934426.
43. Wu G, van der Helm FC, Veeger HE, Makhsous M, Van Roy P, Anglin C, et al. ISB recommendation on definitions of joint coordinate systems of various joints for the reporting of human joint motion--Part II: shoulder, elbow, wrist and hand. J Biomech. 2005;38(5):981-92. PubMed PMID: 15844264.

**List of Abbreviations**

4SST – Four Square Step Test

6-min walk – 6-Minute Walk Test

AmpPRO – Amputee Mobility Predictor

ANOVA – Analysis of Variance

CMS - Centers for Medicare & Medicaid Services

CPCR – Center for Performance and Clinical Research

ESR – Energy Storing and Returning

FIM – Functional Independence Measure

HAI – Hill Assessment Index

ISB – International Society of Biomechanics

OEF – Operation Enduring Freedom

OIF – Operation Iraqi Freedom

OND – Operation New Dawn

OPUS – Orthotics and Prosthetics User Survey

PEQ – Prosthetic Evaluation Questionnaire

ROC – Receiver Operating Characteristic

SACH – Solid Ankle Cushion Heel

SAFE – Stationary Attachment Flexible Endoskeletal

SAI – Stair Assessment Index

SAM – Step Activity Monitor

SF-12 – Short Form-12

TFA – Transfemoral Amputation

TTA – Transtibial Amputation

TUG – Timed-Up-And-Go

VANYHHS – Veterans Affairs New York Harbor Healthcare System

VAS – Visual Analog Scale

WRNMMC – Walter Reed National Military Medical Center

**Data Management**

A. Data Management

**Data Management between sites:**

*Data Management Between Sites:*
A one-day training kick off meeting will be held at the VA NYHHS prior to study initiation to review and train research staff on the research protocol for each site. This will include comprehensive training of each aspect of the protocol, including fittings, device trainings, data collection and recording, data analysis, and sharing of results.

An additional training will take place between the NYHHS and WRNMMC to review the biomechanical gait analysis between sites. While an interlab reliability and validity study has not been conducted between WRNMMC and VA NYHHS at this time, a proposal to conduct this study has been developed and preliminary funding has been identified. We plan to conduct this investigation prior study initiation and expect positive findings by implementing the recommendations provided in the military treatment facility interlab reliability study (Data was presented at Gait and Clinical Movement Analysis Society 2015 Meeting, March 18-21, Portland, OR). We plan to use identical marker sets and analysis, including identical anatomical segment definitions, to reduce variability between the labs to ensure consistent data collection and analysis.

Monthly video conferences between all sites will be conducted to ensure compliance, review the study protocol/results, and mitigate any problems. Annual meetings will take place prior to the start of each year to review data and ensure compliance. Interim analysis of data will take place between the site PIs at 25%, 50%, 75% enrollment. Data analysis of the remaining subjects will be initiated after the final subject has completed the protocol. All data shared amongst team members for analysis and interpretation will be de-identified and will not include PHI. De-identified data will also be entered in a Clinical Trials Database (CTDB). The CTDB is a protocol and data management system used to assist investigators to capture and manage de-identified data. All data will be stored in an access-controlled database with end-to-end government grade encryption. Each user must have a unique username and password to log in to the database. User passwords meet industry-standard strength requirements and expire every 90 days. For each protocol, the PI will have the responsibility of granting access to members of the protocol team. The entire database structure is hosted at a secure US data center that specializes in serving Federal agencies' IT projects requiring high levels of security compliance certification. Exchange of de-identified data between sites will occur through the CTDB or a similar secure manner.

**Identifiers:**

The following unique identifiers will be collected:

- Name
- Telephone number
- Social Security Number (for the Informed Consent Form)

Any identifiers will be kept within the master list secured in a password protected file on a secured government computer at the site the participant is enrolled, VA NYHHS or WRNMMC. Only the PI and those on the protocol responsible for contacting the participants for follow-up appointments will have access to the document. All documents will be coded with a unique study ID immediately upon receiving signed consent and the subject is enrolled into the study. Unique IDs with include a site indicator, such as WR001 and NY001, to not duplicate ID numbers across sites, and will not include any part of the participant's social security number. No personal identifying information will be used to label the data. Research records will be kept in a data

collection folder, with no personal identifying information written on the documents. Research records will be labeled with only the subject ID number.

**Confidentiality**

All research documents will be coded with a unique study ID immediately upon receiving signed consent and the participant is enrolled into the study. Unique IDs with include a site indicator, such as NY001 and WR001, to not duplicate ID numbers across sites, and will not include any part of the participant's social security number. No personal identifying information will be used to label the data. A single master document with names of subjects and corresponding coding will be secured in the PI's office at the site the subject is enrolled. The data will be analyzed and stored in secure, password-protected laboratory computers or VA/DoD servers, accessible only to study personnel. All hard-copies of data collection forms will be secured in a locked file drawer and accessible only by study personnel.

In any reports or presentations, subjects will be referred to by code and not by name. Any data that may be published will not reveal the identity of the subjects. In the interest of public safety, subject information will be provided to the federal and state regulatory agencies as required by law. Applicable regulations note the possibility that the U.S. Army Medical Research and Material Command (USAMRMC) may inspect the records.

Video recordings of gait do not record identifiable views, such as the face, of the subjects. The technology only records the locations of reflective markers placed on specific anatomical positions of the participant. No personal identifying information will be used to label the data. Research records will be kept in a data collection folder, with no personal identifying information written on the documents. Upon entering the study, subjects will be assigned a unique subject ID number that is not any part of their social security number. Research records will be labeled with only the subject ID number.

**Disposition of Data**

The master list will be secured in a password protected file on a secured government computer at the site the participant is enrolled and will be destroyed at the time of study closure. The de-identified data will be analyzed and stored in password-protected laboratory computers, accessible only to study personnel at the site of enrollment. No personal identifying information will be included in the electronic file on in the name of the file. This de-identified electronic data will be stored on these password-protected laboratory computers indefinitely. The site Institutional Review Board has approved indefinite storage of de-identified data with the hope that new questions that arise may be able to be answered through previously collected data.

Hard copy research records will be also be labeled with only the participant's ID number, will be secured in a locked file drawer and accessible only by study personnel at the site of enrollment. These files will be destroyed three years after study closure.

**Sharing Study Results**

At the time of completion, the participant may request any/all of the data collected during the study, and it will be their choice to share it with their rehabilitative care team. The research team does not believe that choosing to not share the results obtained through the evaluations will greatly impact the medical care provided to the participants, to a point that it will outweigh the negative impact on the study. Participants may opt to have the training sessions documented in their medical record. This decision will be indicated in the informed consent form, so the rehabilitation team will have visibility of the training plan and performance in these instances.

B. No human biological samples will be collected for this study
