# Supplementary material for: Toward evidence-based prescription of prosthetic ankle-foot devices: A multisite randomized crossover trial identifying performance-based, patient-reported, and biomechanical parameters sensitive to device type
Source: PLoS One. 2026 Jul 2;21(7):e0352644. doi: 10.1371/journal.pone.0352644 (PMC13327263; doi:10.1371/journal.pone.0352644)
Supplement: S1 Table — Linear mixed model estimates and paired comparisons are shown for the 6-minute walk test (6MWT), Amputee Mobility Predictor (AmpPro), Timed Up and Go (TUG), Four Square Step Test (4SST), Stair Assessment Index (SAI), and Hill Assessment Index (HAI) measures. (DOCX) [file pone.0352644.s002.docx]

| **S1 Table. Performance-based outcomes for each ankle-foot device type.** Linear mixed model estimates and paired comparisons are shown for the 6-minute walk test (6MWT), Amputee Mobility Predictor (AmpPro), Timed Up and Go (TUG), Four Square Step Test (4SST), Stair Assessment Index (SAI), and Hill Assessment Index (HAI) measures. | | | | |
| --- | --- | --- | --- | --- |
|  |  |  | *Linear Mixed Model (n=91)* | |
| Parameter | Mean (SD) | *Paired Outcomes* | *Estimate (SE)* | *p-value* |
| **6MWT (m)** |  |  |  |  |
| ESR | 426.2 (121.1) | ESR-ART | 6.7 (5.6) | 0.46 |
| ART | 419.7 (120.0) | ESR-PWR | -1.7 (5.6) | 0.95 |
| PWR | 427.3 (116.4) | ART-PWR | -8.3 (5.6) | 0.30 |
| **AmpPRO** |  |  |  |  |
| ESR | 41.4 (4.3) | ESR-ART | 0.3 (0.3) | 0.62 |
| ART | 41.2 (4.7) | ESR-PWR | 0.3 (0.3) | 0.62 |
| PWR | 41.1 (4.4) | ART-PWR | -0.0 (0.3) | 1.00 |
| **TUG (s)** |  |  |  |  |
| ESR | 8.9 (3.2) | ESR-ART | -0.5 (0.1) | **0.00** |
| ART | 9.3 (3.4) | ESR-PWR | -0.3 (0.1) | **0.04** |
| PWR | 9.3 (3.3) | ART-PWR | 0.1 (0.1) | 0.74 |
| **4SST (s)** |  |  |  |  |
| ESR | 11.1 (5.1) | ESR-ART | -0.2 (0.3) | 0.86 |
| ART | 11.2 (5.7) | ESR-PWR | -0.5 (0.3) | 0.25 |
| PWR | 11.7 (5.1) | ART-PWR | -0.3 (0.3) | 0.54 |
| **SAI Ascent** | |  |  |  |
| ESR | 10.6 (3.1) | ESR-ART | 0.1 (0.3) | 0.85 |
| ART | 10.5 (3.2) | ESR-PWR | 0.2 (0.3) | 0.67 |
| PWR | 10.4 (3.1) | ART-PWR | 0.1 (0.3) | 0.95 |
| **SAI Descent** | |  |  |  |
| ESR | 10.1 (3.5) | ESR-ART | 0.3 (0.3) | 0.52 |
| ART | 9.9 (3.6) | ESR-PWR | 0.4 (0.3) | 0.31 |
| PWR | 9.8 (3.7) | ART-PWR | 0.1 (0.3) | 0.92 |
| **HAI Ascent** | |  |  |  |
| ESR | 10.1 (1.5) | ESR-ART | -0.1 (0.2) | 0.78 |
| ART | 10.2 (1.4) | ESR-PWR | -0.1 (0.2) | 0.93 |
| PWR | 10.2 (1.6) | ART-PWR | 0.1 (0.2) | 0.95 |
| **HAI Descent** | |  |  |  |
| ESR | 10.1 (1.2) | ESR-ART | -0.1 (0.1) | 0.92 |
| ART | 10.2 (1.5) | ESR-PWR | 0.1 (0.1) | 0.87 |
| PWR | 10.1 (1.7) | ART-PWR | 0.1 (0.1) | 0.64 |

**Abbreviations:** 6MWT: 6-minute walk test (meters); AmpPro: Amputee Mobility Predictor; TUG: Timed Up and Go (seconds); 4SST: Four Square Step Test (seconds); SAI: Stair Assessment Index; HAI: Hill Assessment Index; ESR: energy storing and returning; ART: articulating; PWR: powered.
