# Supplementary material for: Toward evidence-based prescription of prosthetic ankle-foot devices: A multisite randomized crossover trial identifying performance-based, patient-reported, and biomechanical parameters sensitive to device type
Source: PLoS One. 2026 Jul 2;21(7):e0352644. doi: 10.1371/journal.pone.0352644 (PMC13327263; doi:10.1371/journal.pone.0352644)
Supplement: S2 Table — Linear mixed model estimates (SE) and paired comparisons are shown for the 12-Item Short Form Health Survey (SF-12), Prosthesis Evaluation Questionnaire (PEQ), and Orthotics and Prosthetics Users’ Survey (OPUS) measures. (DOCX) [file pone.0352644.s003.docx]

| **S2 Table. Patient-reported outcomes for each ankle-foot device type.** Linear mixed model estimates (SE) and paired comparisons are shown for the 12-Item Short Form Health Survey (SF-12), Prosthesis Evaluation Questionnaire (PEQ), and Orthotics and Prosthetics Users' Survey (OPUS) measures. | | | | |
| --- | --- | --- | --- | --- |
|  |  |  | *Linear Mixed Model (n=91)* | |
| *Parameter* | *Mean (SD)* | *Paired Outcomes* | *Estimate (SE)* | *p-value* |
| **SF-12 Physical** | |  |  |  |
| ESR | 42.2 (9.8) | ESR-ART | -0.6 (0.7) | 0.66 |
| ART | 42.7 (9.0) | ESR-PWR | -1.2 (0.7) | 0.20 |
| PWR | 43.4 (9.8) | ART-PWR | -0.6 (0.7) | 0.67 |
| **SF-12 Mental** | |  |  |  |
| ESR | 51.6 (10.0) | ESR-ART | -0.6 (0.7) | 0.65 |
| ART | 52.5 (9.7) | ESR-PWR | -0.3 (0.7) | 0.91 |
| PWR | 51.8 (10.5) | ART-PWR | 0.3 (0.7) | 0.89 |
| **PEQ Satisfaction** | |  |  |  |
| ESR | 83.4 (15.8) | ESR-ART | 9.5 (2.8) | **0.00** |
| ART | 74.2 (20.5) | ESR-PWR | 12.34 (2.8) | **0.00** |
| PWR | 71.6 (23.6) | ART-PWR | 2.9 (2.8) | 0.56 |
| **PEQ Ambulation** | |  |  |  |
| ESR | 79.4 (16.9) | ESR-ART | 0.2 (1.6) | 0.99 |
| ART | 79.2 (16.0) | ESR-PWR | 1.4 (1.6) | 0.66 |
| PWR | 78.3 (18.6) | ART-PWR | 1.2 (1.6) | 0.74 |
| **PEQ Appearance** | |  |  |  |
| ESR | 80.4 (17.4) | ESR-ART | -0.9 (2.0) | 0.90 |
| ART | 81.2 (16.2) | ESR-PWR | 3.2 (2.0) | 0.23 |
| PWR | 77.2 (17.1) | ART-PWR | 4.1 (2.0) | 0.09 |
| **PEQ Frustration** | |  |  |  |
| ESR | 81.7 (23.2) | ESR-ART | 8.0 (3.2) | **0.04** |
| ART | 74.3 (27.1) | ESR-PWR | 12.0 (3.2) | **0.00** |
| PWR | 70.3 (28.89) | ART-PWR | 4.0 (3.2) | 0.42 |
| **PEQ Perceived Response** | |  |  |  |
| ESR | 91.3 (11.3) | ESR-ART | 1.8 (1.3) | 0.39 |
| ART | 89.7 (14.0) | ESR-PWR | 4.1 (1.3) | **0.00** |
| PWR | 87.7 (14.9) | ART-PWR | 2.3 (1.3) | 0.19 |
| **PEQ Residual Limb Health** | |  |  |  |
| ESR | 79.2 (16.3) | ESR-ART | -0.9 (1.60) | 0.85 |
| ART | 80.2 (16.5) | ESR-PWR | 0.2 (1.60) | 0.99 |
| PWR | 79.4 (16.6) | ART-PWR | 1.1 (1.60) | 0.78 |
| **PEQ Social Burden** | |  |  |  |
| ESR | 86.0 (17.5) | ESR-ART | -0.6 (1.8) | 0.94 |
| ART | 87.0 (14.1) | ESR-PWR | 2.1 (1.8) | 0.48 |
| PWR | 84.3 (18.9) | ART-PWR | 2.7 (1.8) | 0.30 |
| **PEQ Sounds** | |  |  |  |
| ESR | 75.2 (26.5) | ESR-ART | 2.2 (3.5) | 0.82 |
| ART | 73.2 (26.2) | ESR-PWR | 27.7 (3.5) | **0.000** |
| PWR | 48.4 (34.2) | ART-PWR | 25.5 (3.5) | **0.000** |
| **PEQ Utility** | |  |  |  |
| ESR | 81.3 (13.2) | ESR-ART | 6.5 (1.9) | **0.00** |
| ART | 75.0 (17.1) | ESR-PWR | 10.45 (1.9) | **0.00** |
| PWR | 71.3 (17.3) | ART-PWR | 4.0 (1.9) | 0.10 |
| **PEQ Well Being** | |  |  |  |
| ESR | 82.2 (16.4) | ESR-ART | 2.2 (1.5) | 0.31 |
| ART | 79.4 (19.8) | ESR-PWR | 1.2 (1.5) | 0.72 |
| PWR | 81.0 (16.0) | ART-PWR | -1.0 (1.5) | 0.77 |
| **OPUS Satisfaction with Devices** | |  |  |  |
| ESR | 42.5 (7.0) | ESR-ART | 0.5 (0.8) | 0.83 |
| ART | 42.2 (7.1) | ESR-PWR | 1.9 (0.8) | **0.04** |
| PWR | 40.7 (7.3) | ART-PWR | 1.45 (0.8) | 0.15 |
| **OPUS Satisfaction with Services** | |  |  |  |
| ESR | 45.8 (5.1) | ESR-ART | 0.7 (0.6) | 0.39 |
| ART | 45.2 (6.0) | ESR-PWR | -0.3 (0.6) | 0.87 |
| PWR | 46.3 (4.7) | ART-PWR | -1.0 (0.6) | 0.17 |
| **OPUS Functional Status** | |  |  |  |
| ESR | 54.3 (12.9) | ESR-ART | -0.3 (0.7) | 0.92 |
| ART | 54.7 (13.8) | ESR-PWR | -0.4 (0.7) | 0.81 |
| PWR | 55.0 (13.8) | ART-PWR | -0.2 (0.7) | 0.97 |
| **OPUS Quality of Life** | |  |  |  |
| ESR | 67.5 (15.6) | ESR-ART | -1.4 (0.7) | 0.11 |
| ART | 68.9 (16.3) | ESR-PWR | 0.1 (0.7) | 0.99 |
| PWR | 67.6 (15.5) | ART-PWR | 1.5 (0.7) | 0.08 |

**Abbreviations:** SF-12: 12-Item Short Form Health Survey; PEQ: Prosthesis Evaluation Questionnaire; OPUS: Orthotics and Prosthetics Users’ Survey; ESR: energy returning and storing; ART: articulating; PWR: powered
