# Supplementary material for: Toward evidence-based prescription of prosthetic ankle-foot devices: A multisite randomized crossover trial identifying performance-based, patient-reported, and biomechanical parameters sensitive to device type
Source: PLoS One. 2026 Jul 2;21(7):e0352644. doi: 10.1371/journal.pone.0352644 (PMC13327263; doi:10.1371/journal.pone.0352644)
Supplement: S3 Table — Participant characteristics are shown as mean (SD) for continuous variables and n (%) for categorical variables. (DOCX) [file pone.0352644.s004.docx]

| **S3 Table. Demographics of the biomechanical subset.** Participant characteristics are shown as mean (SD) for continuous variables and n (%) for categorical variables. | |
| --- | --- |
| **Biomechanics Subset Sample (n=29)** | |
| **Age (years)** |  |
| Mean (SD) | 50.6 (12.5) |
| **Height (cm)** |  |
| Mean (SD) | 182.2 (7.9) |
| **Body Mass (kg)** |  |
| Mean (SD) | 90.9 (18.7) |
| **Sex** n (%) |  |
| Men | 25 (86.2) |
| Women | 4 (13.8) |
| **Etiology of Limb Loss** n (%) |  |
| Trauma | 22 (75.9) |
| Vascular Disease/Diabetes | 4 (13.8) |
| Cancer | 3 (10.3) |
| **Time Since Limb Loss (months)** |  |
| Mean (SD) | 133.7 (152.2) |
| **Clinician-derived K-Level** n (%) |  |
| K3 | 9 (31.0) |
| K4 | 20 (69.0) |
| **Suspension Type** n (%) |  |
| Pin-Locking | 18 (62.1) |
| Suction | 10 (34.54) |
| Elevated Vacuum | 1 (3.4) |
| **Ethnicity** n (%) |  |
| Not Hispanic or Latino | 27 (93.1) |
| Hispanic or Latino | 2 (6.9) |
| **Race** n (%) |  |
| White | 17 (58.6) |
| Black or African American | 11 (37.9) |
| Identified as more than 1 race | 1 (3.4) |
| **Military Status** n (%) |  |
| Veteran | 19 (65.5) |
| Civilian | 5 (17.2) |
| Active-Duty Service Member | 5 (17.2) |
| **Employment Status** n (%) |  |
| Not currently employed | 12 (41.4) |
| Full time | 12 (41.4) |
| Part time | 5 (17.2) |
| **Living Situation** n (%) |  |
| Lives with others | 21 (72.4) |
| Lives alone | 8 (27.6) |
| **Relationship Status** n (%) |  |
| Married | 13 (44.8) |
| Divorced | 7 (24.1) |
| Never Married | 6 (20.7) |
| Separated | 2 (6.9) |
| Widowed | 1 (3.4) |

**Abbreviations:** K-level: Medicare functional classification level.
