# Supplementary material for: Toward evidence-based prescription of prosthetic ankle-foot devices: A multisite randomized crossover trial identifying performance-based, patient-reported, and biomechanical parameters sensitive to device type
Source: PLoS One. 2026 Jul 2;21(7):e0352644. doi: 10.1371/journal.pone.0352644 (PMC13327263; doi:10.1371/journal.pone.0352644)
Supplement: S4 Table — Linear mixed model estimates (SE) and paired comparisons are shown for the 19 unique biomechanical parameters at the ankle, knee, and hip across the three walking speeds for the subset of 29 participants. Percent change from control indicates differences relative to control values. The final column identifies which device had the lowest percent change from control for each parameter. Units are included where relevant. (DOCX) [file pone.0352644.s005.docx]

**S4 Table. Biomechanical outcomes across ankle-foot devices at each speed.** Linear mixed model estimates (SE) and paired comparisons are shown for the 19 unique biomechanical parameters at the ankle, knee, and hip across the 3 walking speeds for the subset of 29 participants. Percent change from control indicates differences relative to control values. The final column identifies which device had the lowest percent change from control for each parameter. Units are included where relevant.

| ***Joint*** | ***Parameter*** | ***Speed (m/s)*** | ***Type*** | ***Mean (SD)*** | ***Control Mean (SD)*** | ***% Change from Control*** | ***Paired Outcomes*** | ***Estimate (SE)*** | ***p- value*** | **Device Lowest % Change from Control** |
| --- | --- | --- | --- | --- | --- | --- | --- | --- | --- | --- |
| Ankle | Maximum DF: Stance (^o^) | 1.0 | ESR | 19.44 (3.93) | 18.24 (1.96) | 6.61 | ESR_ART | 1.14 (0.87) | 0.39 | None |
|  |  |  | ART | 18.29 (3.89) |  | 0.30 | ART_PWR | 1.74 (0.85) | 0.11 |  |
|  |  |  | PWR | 16.55 (3.20) |  | -9.24 | ESR_PWR | 2.88 (0.87) | **0.00** |  |
|  |  | 1.3 | ESR | 20.29 (4.10) | 17.37 (2.29) | 16.78 | ESR_ART | 1.34 (0.88) | 0.29 | None |
|  |  |  | ART | 18.95 (3.87) |  | 9.09 | ART_PWR | 1.68 (0.88) | 0.15 |  |
|  |  |  | PWR | 17.27 (3.29) |  | -0.60 | ESR_PWR | 3.02 (0.88) | **0.00** |  |
|  |  | 1.5 | ESR | 20.84 (4.07) | 16.71 (2.62) | 24.72 | ESR_ART | 1.29 (0.87) | 0.31 | None |
|  |  |  | ART | 19.54 (3.83) |  | 16.95 | ART_PWR | 2.04 (0.86) | 0.06 |  |
|  |  |  | PWR | 17.53 (3.32) |  | 4.92 | ESR_PWR | 3.33 (0.86) | **0.00** |  |
| Ankle | Minimum PF: Stance (^o^) | 1.0 | ESR | 3.27 (4.86) | -2.84 (3.02) | 215.23 | ESR_ART | 3.96 (0.99) | **0.00** | PWR, ART |
|  |  |  | ART | -0.72 (4.26) |  | 74.58 | ART_PWR | 2.21 (0.98) | 0.07 |  |
|  |  |  | PWR | -2.93 (2.99) |  | -3.18 | ESR_PWR | 6.17 (0.99) | **0.00** |  |
|  |  | 1.3 | ESR | 2.49 (5.24) | -3.95 (3.69) | 162.99 | ESR_ART | 4.09 (1.03) | **0.00** | PWR, ART |
|  |  |  | ART | -1.60 (4.33) |  | 59.41 | ART_PWR | 2.33 (1.03) | 0.07 |  |
|  |  |  | PWR | -3.93 (3.08) |  | 0.31 | ESR_PWR | 6.42 (1.03) | **0.00** |  |
|  |  | 1.5 | ESR | 2.36 (5.36) | -5.53 (4.53) | 142.63 | ESR_ART | 3.93 (1.00) | **0.00** | PWR |
|  |  |  | ART | -1.66 (4.03) |  | 70.02 | ART_PWR | 3.08 (0.99) | **0.01** |  |
|  |  |  | PWR | -4.65 (3.07) |  | 15.91 | ESR_PWR | 7.00 (0.99) | **0.00** |  |
| Ankle | ROM (^o^) | 1 | ESR | 16.17 (3.79) | 21.07 (2.04) | -23.27 | ESR_ART | -2.84 (0.80) | **0.00** | PWR, ART |
|  |  |  | ART | 19.01 (4.23) |  | -9.79 | ART_PWR | -0.47 (0.79) | 0.83 |  |
|  |  |  | PWR | 19.48 (2.14) |  | -7.57 | ESR_PWR | -3.31 (0.80) | **0.00** |  |
|  |  | 1.3 | ESR | 17.80 (4.30) | 21.32 (2.22) | -16.49 | ESR_ART | -2.75 (0.91) | **0.01** | PWR, ART |
|  |  |  | ART | 20.55 (4.56) |  | -3.59 | ART_PWR | -0.65 (0.91) | 0.76 |  |
|  |  |  | PWR | 21.20 (2.46) |  | -0.55 | ESR_PWR | -3.40 (0.91) | **0.00** |  |
|  |  | 1.5 | ESR | 18.49 (4.72) | 22.24 (2.60) | -16.86 | ESR_ART | -2.60 (0.94) | 0.02 | None |
|  |  |  | ART | 21.20 (4.59) |  | -4.66 | ART_PWR | -1.07 (0.93) | 0.49 |  |
|  |  |  | PWR | 22.18 (2.57) |  | -0.26 | ESR_PWR | -3.67 (0.93) | **0.00** |  |
| Ankle | Peak PF: Loading Response (^o^) | 1 | ESR | 3.42 (4.85) | -1.37 (2.62) | 349.71 | ESR_ART | 4.06 (1.00) | **0.00** | ART, PWR |
|  |  |  | ART | -0.69 (4.25) |  | 49.53 | ART_PWR | 1.71 (0.99) | 0.20 |  |
|  |  |  | PWR | -2.40 (3.33) |  | -75.01 | ESR_PWR | 5.77 (1.00) | **0.00** |  |
|  |  | 1.3 | ESR | 2.50 (5.24) | -0.69 (2.46) | 462.38 | ESR_ART | 3.97 (1.05) | **0.00** | ART, PWR |
|  |  |  | ART | -1.47 (4.29) |  | -113.33 | ART_PWR | 1.81 (1.05) | 0.21 |  |
|  |  |  | PWR | -3.28 (3.57) |  | -375.43 | ESR_PWR | 5.78 (1.05) | **0.00** |  |
|  |  | 1.5 | ESR | 2.37 (5.36) | 0.36 (2.32) | 551.60 | ESR_ART | 3.77 (1.01) | **0.00** | None |
|  |  |  | ART | -1.51 (3.88) |  | -516.04 | ART_PWR | 2.63 (1.00) | 0.03 |  |
|  |  |  | PWR | -4.05 (3.47) |  | -1216.57 | ESR_PWR | 6.40 (1.00) | **0.00** |  |
| Ankle | Peak PF: PreSwing (^o^) | 1 | ESR | 9.79 (3.37) | -0.99 (3.92) | 1091.99 | ESR_ART | 0.62 (0.78) | 0.70 | PWR |
|  |  |  | ART | 9.20 (2.97) |  | 1031.66 | ART_PWR | 8.49 (0.77) | **0.00** |  |
|  |  |  | PWR | 0.71 (4.04) |  | 171.45 | ESR_PWR | 9.12 (0.78) | **0.00** |  |
|  |  | 1.3 | ESR | 10.23 (3.52) | -3.21 (4.51) | 418.65 | ESR_ART | 0.71 (0.84) | 0.68 | PWR |
|  |  |  | ART | 9.51 (2.88) |  | 396.45 | ART_PWR | 9.79 (0.84) | **0.00** |  |
|  |  |  | PWR | -0.28 (4.39) |  | 91.22 | ESR_PWR | 10.51 (0.84) | **0.00** |  |
|  |  | 1.5 | ESR | 10.84 (3.31) | -5.17 (5.03) | 309.42 | ESR_ART | 0.73 (0.82) | 0.65 | PWR |
|  |  |  | ART | 10.10 (2.68) |  | 295.23 | ART_PWR | 11.06 (0.81) | **0.00** |  |
|  |  |  | PWR | -0.92 (4.25) |  | 82.31 | ESR_PWR | 11.78 (0.81) | **0.00** |  |
| Ankle | PF Moment: Loading Response (Nm/kg) | 1 | ESR | -0.16 (0.12) | -0.18 (0.05) | 14.31 | ESR_ART | 0.11 (0.02) | **0.00** | ESR |
|  |  |  | ART | -0.26 (0.11) |  | -42.53 | ART_PWR | 0.04 (0.02) | 0.15 |  |
|  |  |  | PWR | -0.30 (0.10) |  | -65.23 | ESR_PWR | 0.15 (0.02) | **0.00** |  |
|  |  | 1.3 | ESR | -0.21 (0.16) | -0.25 (0.06) | 15.75 | ESR_ART | 0.12 (0.03) | **0.00** | ESR |
|  |  |  | ART | -0.33 (0.12) |  | -32.20 | ART_PWR | 0.03 (0.03) | 0.58 |  |
|  |  |  | PWR | -0.36 (0.13) |  | -43.19 | ESR_PWR | 0.15 (0.03) | **0.00** |  |
|  |  | 1.5 | ESR | -0.23 (0.17) | -0.28 (0.06) | 17.80 | ESR_ART | 0.12 (0.03) | **0.00** | ESR |
|  |  |  | ART | -0.36 (0.14) |  | -27.46 | ART_PWR | 0.06 (0.03) | 0.11 |  |
|  |  |  | PWR | -0.41 (0.16) |  | -47.11 | ESR_PWR | 0.19 (0.03) | **0.00** |  |
| Ankle | DF Moment: Terminal Stance (Nm/kg) | 1 | ESR | 1.39 (0.32) | 1.32 (0.12) | 5.67 | ESR_ART | 0.16 (0.06) | 0.02 | None |
|  |  |  | ART | 1.23 (0.23) |  | -6.56 | ART_PWR | 0.08 (0.06) | 0.37 |  |
|  |  |  | PWR | 1.16 (0.11) |  | -12.34 | ESR_PWR | 0.24 (0.06) | **0.00** |  |
|  |  | 1.3 | ESR | 1.48 (0.33) | 1.47 (0.13) | 0.30 | ESR_ART | 0.18 (0.06) | **0.01** | ESR |
|  |  |  | ART | 1.29 (0.25) |  | -12.26 | ART_PWR | 0.08 (0.06) | 0.41 |  |
|  |  |  | PWR | 1.22 (0.13) |  | -17.38 | ESR_PWR | 0.26 (0.06) | **0.00** |  |
|  |  | 1.5 | ESR | 1.49 (0.33) | 1.56 (0.14) | -3.93 | ESR_ART | 0.19 (0.06) | **0.01** | ESR |
|  |  |  | ART | 1.31 (0.27) |  | -15.83 | ART_PWR | 0.07 (0.06) | 0.53 |  |
|  |  |  | PWR | 1.24 (0.14) |  | -20.09 | ESR_PWR | 0.26 (0.06) | **0.00** |  |
| Ankle | Peak Moment (Nm/kg) | 1 | ESR | 1.41 (0.32) | 1.33 (0.12) | 5.99 | ESR_ART | 0.16 (0.06) | 0.02 | None |
|  |  |  | ART | 1.25 (0.21) |  | -5.93 | ART_PWR | 0.08 (0.05) | 0.30 |  |
|  |  |  | PWR | 1.17 (0.11) |  | -12.08 | ESR_PWR | 0.24 (0.06) | **0.00** |  |
|  |  | 1.3 | ESR | 1.49 (0.33) | 1.47 (0.13) | 1.03 | ESR_ART | 0.18 (0.06) | **0.01** | ESR |
|  |  |  | ART | 1.31 (0.23) |  | -10.95 | ART_PWR | 0.09 (0.06) | 0.24 |  |
|  |  |  | PWR | 1.22 (0.13) |  | -17.18 | ESR_PWR | 0.27 (0.06) | **0.00** |  |
|  |  | 1.5 | ESR | 1.51 (0.34) | 1.56 (0.14) | -3.24 | ESR_ART | 0.19 (0.06) | **0.01** | ESR |
|  |  |  | ART | 1.32 (0.26) |  | -15.11 | ART_PWR | 0.08 (0.06) | 0.43 |  |
|  |  |  | PWR | 1.25 (0.14) |  | -19.97 | ESR_PWR | 0.27 (0.06) | **0.00** |  |
| Ankle | Absorption: Loading Response (W/kg) | 1 | ESR | -0.27 (0.19) | -0.31 (0.11) | 11.12 | ESR_ART | 0.02 (0.04) | 0.76 | None |
|  |  |  | ART | -0.30 (0.15) |  | 3.28 | ART_PWR | 0.05 (0.03) | 0.29 |  |
|  |  |  | PWR | -0.35 (0.13) |  | -13.74 | ESR_PWR | 0.08 (0.04 | 0.08 |  |
|  |  | 1.3 | ESR | -0.34 (0.22) | -0.50 (0.14) | 32.48 | ESR_ART | 0.11 (0.05) | 0.07 | None |
|  |  |  | ART | -0.44 (0.18) |  | 11.31 | ART_PWR | 0.06 (0.05) | 0.40 |  |
|  |  |  | PWR | -0.51 (0.19) |  | -0.92 | ESR_PWR | 0.17 (0.05) | **0.00** |  |
|  |  | 1.5 | ESR | -0.41 (0.20) | -0.65 (0.18) | 36.82 | ESR_ART | 0.10 (0.05) | 0.13 | PWR |
|  |  |  | ART | -0.51 (0.21) |  | 21.64 | ART_PWR | 0.16 (0.05) | **0.01** |  |
|  |  |  | PWR | -0.67 (0.22) |  | -3.49 | ESR_PWR | 0.26 (0.05) | **0.00** |  |
| Ankle | Generation: PreSwing (W/kg) | 1 | ESR | 1.49 (0.48) | 1.80 (0.29) | -16.94 | ESR_ART | 0.47 (0.11) | **0.00** | PWR |
|  |  |  | ART | 1.02 (0.34) |  | -43.32 | ART_PWR | -0.81 (0.11) | **0.00** |  |
|  |  |  | PWR | 1.83 (0.36) |  | 2.01 | ESR_PWR | -0.34 (0.11) | **0.01** |  |
|  |  | 1.3 | ESR | 2.00 (0.63) | 2.57 (0.42) | -22.15 | ESR_ART | 0.67 (0.15) | **0.00** | PWR |
|  |  |  | ART | 1.33 (0.42) |  | -48.21 | ART_PWR | -1.22 (0.15) | **0.00** |  |
|  |  |  | PWR | 2.55 (0.60) |  | -0.53 | ESR_PWR | -0.56 (0.15) | **0.00** |  |
|  |  | 1.5 | ESR | 2.32 (0.79) | 3.14 (0.50) | -26.06 | ESR_ART | 0.83 (0.17) | **0.00** | PWR |
|  |  |  | ART | 1.51 (0.50) |  | -51.85 | ART_PWR | -1.56 (0.17) | **0.00** |  |
|  |  |  | PWR | 3.07 (0.74) |  | -2.41 | ESR_PWR | -0.73 (0.17) | **0.00** |  |
| Ankle | Peak Power (W/kg) | 1 | ESR | 1.49 (0.48) | 1.8 (0.29) | -16.94 | ESR_ART | 0.47 (0.11) | **0.00** | PWR |
|  |  |  | ART | 1.02 (0.34) |  | -43.32 | ART_PWR | -0.81 (0.11) | **0.00** |  |
|  |  |  | PWR | 1.83 (0.36) |  | 2.01 | ESR_PWR | -0.34 (0.11) | **0.01** |  |
|  |  | 1.3 | ESR | 2.00 (0.63) | 2.57 (0.42) | -22.15 | ESR_ART | 0.67 (0.15) | **0.00** | PWR |
|  |  |  | ART | 1.33 (0.42) |  | -48.21 | ART_PWR | -1.22 (0.15) | **0.00** |  |
|  |  |  | PWR | 2.55 (0.60) |  | -0.53 | ESR_PWR | -0.56 (0.15) | **0.00** |  |
|  |  | 1.5 | ESR | 2.32 (0.79) | 3.14 (0.50) | -26.06 | ESR_ART | 0.83 (0.17) | **0.00** | PWR |
|  |  |  | ART | 1.51 (0.50) |  | -51.85 | ART_PWR | -1.56 (0.17) | **0.00** |  |
|  |  |  | PWR | 3.07 (0.74) |  | -2.41 | ESR_PWR | -0.73 (0.17) | **0.00** |  |
| Knee | Minimum Extension: Stance (^o^) | 1 | ESR | 6.54 (6.48) | 3.29 (2.93) | 98.93 | ESR_ART | 3.99 (1.34) | **0.01** | ART, PWR |
|  |  |  | ART | 2.31 (5.81) |  | -29.80 | ART_PWR | 0.17 (1.32) | 0.99 |  |
|  |  |  | PWR | 2.14 (5.91) |  | -34.91 | ESR_PWR | 4.16 (1.34) | **0.01** |  |
|  |  | 1.3 | ESR | 5.21 (7.02) | 3.38 (2.99) | 54.21 | ESR_ART | 3.65 (1.26) | 0.02 | None |
|  |  |  | ART | 1.56 (5.66) |  | -53.87 | ART_PWR | 0.08 (1.26) | 0.99 |  |
|  |  |  | PWR | 1.48 (6.03) |  | -56.09 | ESR_PWR | 3.73 (1.26) | 0.01 |  |
|  |  | 1.5 | ESR | 5.45 (6.67) | 3.18 (3.34) | 71.18 | ESR_ART | 2.90 (1.00) | 0.02 | None |
|  |  |  | ART | 1.91 (5.27) |  | -39.86 | ART_PWR | 1.34 (0.99) | 0.37 |  |
|  |  |  | PWR | 0.99 (6.02) |  | -68.76 | ESR_PWR | 4.24 (0.99) | **0.00** |  |
| Knee | ROM (^o^) | 1 | ESR | 39.95 (6.58) | 43.83 (4.03) | -8.85 | ESR_ART | -3.87 (1.07) | **0.00** | ART, PWR |
|  |  |  | ART | 43.91 (6.69) |  | 0.19 | ART_PWR | -1.90 (1.06) | 0.18 |  |
|  |  |  | PWR | 45.81 (6.34) |  | 4.52 | ESR_PWR | -5.77 (1.07) | **0.00** |  |
|  |  | 1.3 | ESR | 42.69 (5.57) | 42.78 (4.14) | -0.20 | ESR_ART | -3.73 (1.07) | **0.00** | ESR |
|  |  |  | ART | 46.42 (6.91) |  | 8.51 | ART_PWR | -1.22 (1.07) | 0.49 |  |
|  |  |  | PWR | 47.64 (5.75) |  | 11.37 | ESR_PWR | -4.95 (1.07) | **0.00** |  |
|  |  | 1.5 | ESR | 43.16 (4.97) | 42.32 (4.02) | 2.00 | ESR_ART | -2.77 (0.85) | **0.01** | ESR |
|  |  |  | ART | 46.00 (6.67) |  | 8.70 | ART_PWR | -2.54 (0.84) | **0.01** |  |
|  |  |  | PWR | 48.55 (4.91) |  | 14.74 | ESR_PWR | -5.32 (0.84) | **0.00** |  |
| Knee | Flexion: Initial Contact (^o^) | 1 | ESR | 7.79 (7.28) | 4.09 (3.66) | 90.54 | ESR_ART | 2.95 (1.17) | 0.04 | None |
|  |  |  | ART | 4.58 (6.44) |  | 11.93 | ART_PWR | 1.35 (1.16) | 0.48 |  |
|  |  |  | PWR | 3.23 (6.30) |  | -21.03 | ESR_PWR | 4.30 (1.17) | **0.00** |  |
|  |  | 1.3 | ESR | 6.35 (7.74) | 5.00 (3.49) | 27.00 | ESR_ART | 2.47 (1.17) | 0.10 | None |
|  |  |  | ART | 3.87 (6.25) |  | -22.46 | ART_PWR | 1.12 (1.17) | 0.61 |  |
|  |  |  | PWR | 2.76 (6.80) |  | -44.82 | ESR_PWR | 3.59 (1.17) | **0.01** |  |
|  |  | 1.5 | ESR | 6.66 (7.46) | 6.24 (3.62) | 6.64 | ESR_ART | 1.57 (1.04) | 0.30 | None |
|  |  |  | ART | 4.19 (6.56) |  | -32.90 | ART_PWR | 2.52 (1.03) | 0.05 |  |
|  |  |  | PWR | 2.29 (6.62) |  | -63.29 | ESR_PWR | 4.09 (1.03) | **0.00** |  |
| Knee | Maximum Flexion: Loading Response (^o^) | 1 | ESR | 15.68 (6.20) | 15.18 (4.20) | 3.33 | ESR_ART | 2.97 (1.35) | 0.08 | None |
|  |  |  | ART | 12.28 (6.98) |  | -19.07 | ART_PWR | 0.67 (1.32) | 0.87 |  |
|  |  |  | PWR | 11.61 (6.69) |  | -23.47 | ESR_PWR | 3.64 (1.40) | 0.03 |  |
|  |  | 1.3 | ESR | 15.69 (7.75) | 18.18 (4.93) | -13.70 | ESR_ART | 3.24 (1.40) | 0.06 | None |
|  |  |  | ART | 12.45 (7.25) |  | -31.50 | ART_PWR | 0.38 (1.40) | 0.96 |  |
|  |  |  | PWR | 12.07 (6.49) |  | -33.61 | ESR_PWR | 3.62 (1.40) | 0.03 |  |
|  |  | 1.5 | ESR | 16.96 (7.12) | 19.56 (5.49) | -13.29 | ESR_ART | 2.35 (1.21) | 0.14 | None |
|  |  |  | ART | 13.49 (7.48) |  | -31.01 | ART_PWR | 2.45 (1.19) | 0.11 |  |
|  |  |  | PWR | 11.65 (7.06) |  | -40.44 | ESR_PWR | 4.80 (1.19) | **0.00** |  |
| Knee | Knee Moment: Initial Contact (Nm/kg) | 1 | ESR | -0.19 (0.06) | -0.17 (0.07) | -13.81 | ESR_ART | -0.01 (0.01) | 0.62 | None |
|  |  |  | ART | -0.18 (0.09) |  | -6.29 | ART_PWR | -0.02 (0.01) | 0.18 |  |
|  |  |  | PWR | -0.16 (0.07) |  | 8.01 | ESR_PWR | -0.04 (0.01) | 0.03 |  |
|  |  | 1.3 | ESR | -0.28 (0.10) | -0.24 (0.09) | -20.71 | ESR_ART | -0.01 (0.02) | 0.77 | None |
|  |  |  | ART | -0.27 (0.11) |  | -15.60 | ART_PWR | -0.04 (0.02) | 0.05 |  |
|  |  |  | PWR | -0.23 (0.10) |  | 2.61 | ESR_PWR | -0.06 (0.02) | **0.01** |  |
|  |  | 1.5 | ESR | -0.36 (0.12) | -0.26 (0.10) | -36.14 | ESR_ART | -0.02 (0.02) | 0.71 | None |
|  |  |  | ART | -0.34 (0.16) |  | -28.52 | ART_PWR | -0.07 (0.02) | 0.02 |  |
|  |  |  | PWR | -0.27 (0.11) |  | -3.73 | ESR_PWR | -0.09 (0.02) | **0.00** |  |
| Knee | Maximum Absorption: PreSwing (W/kg) | 1 | ESR | -0.70 (0.26) | -0.74 (0.18) | 4.55 | ESR_ART | 0.14 (0.05) | **0.01** | ESR |
|  |  |  | ART | -0.85 (0.38) |  | -14.69 | ART_PWR | 0.10 (0.05) | 0.12 |  |
|  |  |  | PWR | -0.94 (0.32) |  | -27.60 | ESR_PWR | 0.24 (0.05) | **0.00** |  |
|  |  | 1.3 | ESR | -1.16 (0.40) | -1.00 (0.25) | -16.20 | ESR_ART | 0.13 (0.07) | 0.21 | None |
|  |  |  | ART | -1.29 (0.53) |  | -28.68 | ART_PWR | 0.07 (0.07) | 0.58 |  |
|  |  |  | PWR | -1.36 (0.44) |  | -35.98 | ESR_PWR | 0.20 (0.07) | 0.03 |  |
|  |  | 1.5 | ESR | -1.45 (0.46) | -1.21 (0.28) | -19.50 | ESR_ART | 0.18 (0.08) | 0.07 | None |
|  |  |  | ART | -1.64 (0.64) |  | -35.04 | ART_PWR | -0.03 (0.08) | 0.95 |  |
|  |  |  | PWR | -1.61 (0.55) |  | -32.60 | ESR_PWR | 0.16 (0.08) | 0.13 |  |
| Hip | Moment: Initial Contact (Nm/kg) | 1 | ESR | 0.26 (0.12) | 0.21 (0.12) | 25.22 | ESR_ART | 0.00 (0.03) | 0.99 | None |
|  |  |  | ART | 0.27 (0.18) |  | 26.20 | ART_PWR | 0.05 (0.03) | 0.18 |  |
|  |  |  | PWR | 0.22 (0.12) |  | 3.69 | ESR_PWR | 0.04 (0.03) | 0.23 |  |
|  |  | 1.3 | ESR | 0.44 (0.16) | 0.32 (0.15) | 36.56 | ESR_ART | 0.03 (0.03) | 0.74 | None |
|  |  |  | ART | 0.42 (0.20) |  | 28.62 | ART_PWR | 0.09 (0.03) | 0.04 |  |
|  |  |  | PWR | 0.33 (0.16) |  | 1.76 | ESR_PWR | 0.11 (0.03) | **0.01** |  |
|  |  | 1.5 | ESR | 0.57 (0.23) | 0.39 (0.18) | 46.61 | ESR_ART | 0.02 (0.05) | 0.89 | None |
|  |  |  | ART | 0.54 (0.31) |  | 39.93 | ART_PWR | 0.13 (0.05) | 0.02 |  |
|  |  |  | PWR | 0.41 (0.19) |  | 6.92 | ESR_PWR | 0.15 (0.05) | **0.00** |  |
| Hip | Generation: Preswing (W/kg) | 1 | ESR | 0.82 (0.24) | 0.77 (0.17) | 5.93 | ESR_ART | -0.07 (0.04) | 0.23 | None |
|  |  |  | ART | 0.89 (0.28) |  | 15.99 | ART_PWR | 0.08 (0.04) | 0.17 |  |
|  |  |  | PWR | 0.82 (0.25) |  | 6.04 | ESR_PWR | 0.08 (0.04) | 0.99 |  |
|  |  | 1.3 | ESR | 1.29 (0.38) | 1.03 (0.25) | 25.24 | ESR_ART | -0.04 (0.07) | 0.87 | None |
|  |  |  | ART | 1.33 (0.48) |  | 28.76 | ART_PWR | 0.17 (0.07) | 0.06 |  |
|  |  |  | PWR | 1.16 (0.35) |  | 12.18 | ESR_PWR | 0.14 (0.07) | 0.16 |  |
|  |  | 1.5 | ESR | 1.52 (0.44) | 1.25 (0.36) | 22.18 | ESR_ART | -0.06 (0.06) | 0.66 | None |
|  |  |  | ART | 1.59 (0.40) |  | 27.21 | ART_PWR | 0.20 (0.06) | **0.01** |  |
|  |  |  | PWR | 1.39 (0.45) |  | 11.44 | ESR_PWR | 0.15 (0.06) | 0.06 |  |

**Abbreviations:** DF: dorsiflexion; PF: plantarflexion; ROM: range of motion; ESR: energy returning and storing; ART: articulating; PWR: powered
