# Supplementary material for: Toward evidence-based prescription of prosthetic ankle-foot devices: A multisite randomized crossover trial identifying performance-based, patient-reported, and biomechanical parameters sensitive to device type
Source: PLoS One. 2026 Jul 2;21(7):e0352644. doi: 10.1371/journal.pone.0352644 (PMC13327263; doi:10.1371/journal.pone.0352644)
Supplement: Attachment — Submitted filename: Response to Reviewers.docx [file pone.0352644.s008.docx]

We would like to thank the Academic Editor, Dr. Simone Ranaldi, and the reviewers for their time and careful consideration of our manuscript. We are grateful for the constructive feedback provided, which has strengthened the manuscript. We have carefully addressed all journal requirements and reviewer comments below. A revised manuscript with tracked changes and a clean version are included with this submission.

**Response to Journal Requirement 1:** *Please ensure that your manuscript meets PLOS ONE's style requirements, including those for file naming.*

The manuscript has been revised to meet PLOS ONE style requirements, including the title page and file naming conventions. Specifically, the title page was reviewed against the PLOS ONE formatting templates. Two corrections were made to the affiliations: "United States" in Affiliation 9 was standardized to "USA" to match all other affiliations, and a missing comma was added to Affiliation 6 ("Washington, DC, USA"). All other formatting elements, including author byline symbols, affiliation ordering, corresponding author designation, and consortia notation were confirmed to be compliant.

**Response to Journal Requirement 2:** *Ensure that your code is shared in a way that follows best practice and facilitates reproducibility and reuse.*

The statistical analysis code generated by the authors has been deposited in a publicly accessible repository on GitHub (<https://github.com/uconn-scs/prescription_study>) and archived on Zenodo (DOI: 10.5281/zenodo.20215416) along with the study dataset. The repository is freely accessible and is organized to facilitate reproducibility and reuse in accordance with PLOS ONE's code sharing guidelines. The Data Availability Statement has been updated to reflect this.

**Response to Journal Requirement 3:** *Please provide an amended statement that declares *all* the funding or sources of support (whether external or internal to your organization) received during this study.* *Please also include the statement “There was no additional external funding received for this study.” in your updated Funding Statement. Please include your amended Funding Statement within your cover letter. We will change the online submission form on your behalf.*

The Funding Statement has been amended as requested, following PLOS journal guidelines. The updated statement reads:

"Support was provided by the Congressionally Directed Medical Research Programs–Orthotics and Prosthetics Outcomes Research Program (grant W81XWH-17-2-0014; <https://cdmrp.health.mil/>) to JTM, ALP, SLP, JTH, CLD, and LMN. There was no additional external funding received for this study. The funders had no role in study design, data collection and analysis, decision to publish, or preparation of the manuscript."

**Response to Journal Requirements 4 and 5:** *Your entire minimal dataset will need to be made freely accessible if your manuscript is accepted for publication.*

We acknowledge the journal's recommendation to deposit data prior to acceptance and have done so. All data are fully de-identified. All relevant data and code used to generate tables, plots, and perform statistical analyses in this manuscript are available on GitHub (<https://github.com/uconn-scs/prescription_study>) and have been archived on Zenodo (DOI: 10.5281/zenodo.20215416). The repository is freely accessible. The Data Availability Statement in the manuscript has been updated to reflect this.

**Response to Journal Requirement 6:** *Please include captions for your Supporting Information files at the end of your manuscript, and update any in-text citations to match accordingly.*

Supporting information captions have been added to the end of the manuscript as required. The captions are as follows:

**S1 Protocol. Initial institutional review board-approved protocol.**

**S1 Table. Performance-based outcomes for each ankle-foot device type.** Linear mixed model estimates and paired comparisons are shown for the 6-minute walk test (6MWT), Amputee Mobility Predictor (AmpPro), Timed Up and Go test (TUG), Four Square Step Test (4SST), Stair Assessment Index (SAI), and Hill Assessment Index (HAI) measures.

**S2 Table. Patient-reported outcomes for each ankle-foot device type.** Linear mixed model estimates (SE) and paired comparisons are shown for the 12-Item Short Form Health Survey (SF-12), Prosthesis Evaluation Questionnaire (PEQ), and Orthotics and Prosthetics Users' Survey (OPUS) measures.

**S3 Table. Demographics of the biomechanical subset.** Participant characteristics are shown as mean (SD) for continuous variables and n (%) for categorical variables.

**S4 Table. Biomechanical outcomes across ankle-foot devices at each speed.** Linear mixed model estimates (SE) and paired comparisons are shown for the 19 unique biomechanical parameters at the ankle, knee, and hip across the 3 walking speeds for the subset of 29 participants. Percent change from control indicates differences relative to control values. The final column identifies which device had the lowest percent change from control for each parameter. Units are included where relevant.

In-text citations have been reviewed and confirmed to match the supporting information labels above.

**Response to Journal Requirement 7:** *If the reviewer comments include a recommendation to cite specific previously published works, please review and evaluate these publications to determine whether they are relevant and should be cited. There is no requirement to cite these works unless the editor has indicated otherwise.*

No reviewer comments included recommendations to cite specific previously published works. Therefore, no action was required for this item.

**Response to Journal Requirement 8:** *Please review your reference list to ensure that it is complete and correct.*

The reference list was thoroughly reviewed for completeness, accuracy, and retraction status. Each reference was verified through PubMed and Google Scholar. No retracted articles were identified. All references have been confirmed as current and accurate.

**Response to Reviewer #1:**

We thank the reviewer for their careful reading of the manuscript and their overall positive assessment of the study design, statistical approach, and presentation. We note that all three comments appear to reference the S1 Protocol, which is the initial IRB-approved protocol submitted as a required supplement per PLOS ONE's clinical trial reporting guidelines. This document was written prior to study initiation as part of the IRB approval process. While it describes the comprehensive data analysis plan for the entire study across all specific aims, it is a historical document and cannot be modified as part of this revision.

We would like to clarify the relationship between the S1 Protocol and this manuscript. This manuscript represents one of several planned publications from a larger, multi-aim study. The S1 Protocol describes the full scope of the study, including analyses planned at study inception that may be addressed in future manuscripts. As is common in longitudinal, multisite clinical trials, the analytical approach evolved over the course of the study in response to the data and findings as they emerged. The statistical analyses reported in this manuscript are specific to the outcomes presented here and are described in full detail in the Statistical Analyses section of the manuscript.

We address each comment individually below:

**Comment 1:** The reviewer asked about the power considerations, unit differences, and the connection of sample sizes of 120 and 30 to the specific aims as described in the S1 Protocol. These power calculations were developed at study inception to support IRB approval across all specific aims of the larger study. They are not directly relevant to the analyses reported in this manuscript, which uses linear mixed-effects models applied to all available data rather than the power-based sample size targets described in the protocol. The statistical design, including covariates, random effects structure, model assumptions, and multiple comparison adjustments, is described in full in the Statistical Analyses section of this manuscript and is specific to the outcomes reported here.

**Comment 2:** The reviewer noted that the separation units of 3 and 6 referenced in the S1 Protocol are difficult to interpret in the context of the pairwise comparisons reported in Tables S1, S2, and S4. We clarify that the separation units of 3 and 6 in the protocol refer to power calculation assumptions made at study inception for specific aims of the larger study and have no bearing on the analyses or results reported in this manuscript. The pairwise comparisons presented in the supplemental tables are derived from the linear mixed-effects models described in the Statistical Analyses section, with Tukey's Highest Significant Difference used for multiple comparison adjustment. These results stand independently of the protocol's power assumptions.

**Comment 3:** The reviewer asked about ROC curves and AUC values in the context of a logistic regression prediction model referenced in the S1 Protocol. We clarify that prediction modeling is not performed in this manuscript and is outside the scope of the current article. This manuscript focuses on identifying performance-based, patient-reported, and biomechanical parameters sensitive to ankle-foot device type. Linear discriminant analysis was used for dimensionality reduction and to identify discriminative gait features, as described in the Statistical Analyses section and presented in Figures 5 and 6. Prediction modeling, including logistic regression and associated metrics such as ROC curves and AUC, will be addressed in a future manuscript as part of the larger dissemination plan for this study. To clarify this for readers, we have added the following sentence to the Discussion: “Additionally, prediction modeling analyses aimed at identifying the strongest independent predictors of optimal prosthetic prescription outcomes will be addressed in future work as part of the broader dissemination plan for this study.”

**Response to Reviewer #2**

We thank Reviewer 2 for their careful reading of the manuscript and their positive assessment of the study design, statistical approach, and conclusions.
