# Supplementary material for: Toward evidence-based prescription of prosthetic ankle-foot devices: A multisite randomized crossover trial identifying performance-based, patient-reported, and biomechanical parameters sensitive to device type
Source: PLoS One. 2026 Jul 2;21(7):e0352644. doi: 10.1371/journal.pone.0352644 (PMC13327263; doi:10.1371/journal.pone.0352644)
Supplement: Peer review history

# Peer review: Decision Letter 0

**DOI:** 10.1371/journal.pone.0352644.r001

**Authors:** Simone Ranaldi

8 May 2026

PONE-D-26-03461

Toward evidence-based prescription of prosthetic ankle-foot devices: A multisite randomized crossover trial identifying performance-based, patient-reported, and biomechanical parameters sensitive to device type

PLOS One

Dear Dr. Maikos,

Thank you for submitting your manuscript to PLOS ONE. After careful consideration, we feel that it has merit but does not fully meet PLOS ONE’s publication criteria as it currently stands. Therefore, we invite you to submit a revised version of the manuscript that addresses the points raised during the review process.

Please submit your revised manuscript by Jun 22 2026 11:59PM. If you will need more time than this to complete your revisions, please reply to this message or contact the journal office at plosone@plos.org. When you're ready to submit your revision, log on to https://www.editorialmanager.com/pone/ and select the 'Submissions Needing Revision' folder to locate your manuscript file.

Please include the following items when submitting your revised manuscript:

- A letter that responds to each point raised by the academic editor and reviewer(s). You should upload this letter as a separate file labeled 'Response to Reviewers'.
- A marked-up copy of your manuscript that highlights changes made to the original version. You should upload this as a separate file labeled 'Revised Manuscript with Track Changes'.
- An unmarked version of your revised paper without tracked changes. You should upload this as a separate file labeled 'Manuscript'.

If you would like to make changes to your financial disclosure, please include your updated statement in your cover letter. Guidelines for resubmitting your figure files are available below the reviewer comments at the end of this letter.

If applicable, we recommend that you deposit your laboratory protocols in protocols.io to enhance the reproducibility of your results. Protocols.io assigns your protocol its own identifier (DOI) so that it can be cited independently in the future. For instructions see: https://journals.plos.org/plosone/s/submission-guidelines#loc-laboratory-protocols. Additionally, PLOS ONE offers an option for publishing peer-reviewed Lab Protocol articles, which describe protocols hosted on protocols.io. Read more information on sharing protocols at https://plos.org/protocols?utm_medium=editorial-email&utm_source=authorletters&utm_campaign=protocols.

As the corresponding author, your ORCID iD is verified in the submission system and will appear in the published article. PLOS supports the use of ORCID, and we encourage all coauthors to register for an ORCID iD and use it as well. Please encourage your coauthors to verify their ORCID iD within the submission system before final acceptance, as unverified ORCID iDs will not appear in the published article. *Only*  the individual author can complete the verification step; PLOS staff *cannot*  verify ORCID iDs on behalf of authors.

We look forward to receiving your revised manuscript.

Kind regards,

Simone Ranaldi, Ph.D.

Academic Editor

PLOS One

**Journal Requirements:**

1. When submitting your revision, we need you to address these additional requirements.

Please ensure that your manuscript meets PLOS ONE's style requirements, including those for file naming. The PLOS ONE style templates can be found at

https://journals.plos.org/plosone/s/file?id=wjVg/PLOSOne_formatting_sample_main_body.pdf and

https://journals.plos.org/plosone/s/file?id=ba62/PLOSOne_formatting_sample_title_authors_affiliations.pdf

2. Please note that PLOS One has specific guidelines on code sharing for submissions in which author-generated code underpins the findings in the manuscript. In these cases, we expect all author-generated code to be made available without restrictions upon publication of the work. Please review our guidelines at https://journals.plos.org/plosone/s/materials-and-software-sharing#loc-sharing-code and ensure that your code is shared in a way that follows best practice and facilitates reproducibility and reuse.

3. Thank you for stating in your Funding Statement:

Support was provided by the Congressionally Directed Medical Research Programs–Orthotics and Prosthetics Outcomes Research Program (grant W81XWH-17-2-0014). This funding source had no role in the design of this study and did not have any role during its execution, analysis, and interpretation of the data or decision to submit results.

Please provide an amended statement that declares *all* the funding or sources of support (whether external or internal to your organization) received during this study, as detailed online in our guide for authors at http://journals.plos.org/plosone/s/submit-now.  Please also include the statement “There was no additional external funding received for this study.” in your updated Funding Statement.

Please include your amended Funding Statement within your cover letter. We will change the online submission form on your behalf.

4. In the online submission form, you indicated that your data will be submitted to a repository upon acceptance.  We strongly recommend all authors deposit their data before acceptance, as the process can be lengthy and hold up publication timelines. Please note that, though access restrictions are acceptable now, your entire minimal  dataset will need to be made freely accessible if your manuscript is accepted for publication. This policy applies to all data except where public deposition would breach compliance with the protocol approved by your research ethics board. If you are unable to adhere to our open data policy, please kindly revise your statement to explain your reasoning and we will seek the editor's input on an exemption.

5. When completing the data availability statement of the submission form, you indicated that you will make your data available on acceptance. We strongly recommend all authors decide on a data sharing plan before acceptance, as the process can be lengthy and hold up publication timelines. Please note that, though access restrictions are acceptable now, your entire data will need to be made freely accessible if your manuscript is accepted for publication. This policy applies to all data except where public deposition would breach compliance with the protocol approved by your research ethics board. If you are unable to adhere to our open data policy, please kindly revise your statement to explain your reasoning and we will seek the editor's input on an exemption. Please be assured that, once you have provided your new statement, the assessment of your exemption will not hold up the peer review process.

6. Please include captions for your Supporting Information files at the end of your manuscript, and update any in-text citations to match accordingly. Please see our Supporting Information guidelines for more information: http://journals.plos.org/plosone/s/supporting-information.

7. If the reviewer comments include a recommendation to cite specific previously published works, please review and evaluate these publications to determine whether they are relevant and should be cited. There is no requirement to cite these works unless the editor has indicated otherwise.

8. Please review your reference list to ensure that it is complete and correct. If you have cited papers that have been retracted, please include the rationale for doing so in the manuscript text, or remove these references and replace them with relevant current references. Any changes to the reference list should be mentioned in the rebuttal letter that accompanies your revised manuscript. If you need to cite a retracted article, indicate the article’s retracted status in the References list and also include a citation and full reference for the retraction notice.

[Note: HTML markup is below. Please do not edit.]

Reviewers' comments:

Reviewer's Responses to Questions

**Comments to the Author**

1. Is the manuscript technically sound, and do the data support the conclusions?

The manuscript must describe a technically sound piece of scientific research with data that supports the conclusions. Experiments must have been conducted rigorously, with appropriate controls, replication, and sample sizes. The conclusions must be drawn appropriately based on the data presented.

Reviewer #1: Partly

Reviewer #2: Yes

**********

2. Has the statistical analysis been performed appropriately and rigorously?

Reviewer #1: Yes

Reviewer #2: Yes

**********

3. Have the authors made all data underlying the findings in their manuscript fully available?

The PLOS Data policy requires authors to make all data underlying the findings described in their manuscript fully available without restriction, with rare exception (please refer to the Data Availability Statement in the manuscript PDF file). The data should be provided as part of the manuscript or its supporting information, or deposited to a public repository. For example, in addition to summary statistics, the data points behind means, medians and variance measures should be available. If there are restrictions on publicly sharing data—e.g. participant privacy or use of data from a third party—those must be specified.

Reviewer #1: Yes

Reviewer #2: Yes

**********

4. Is the manuscript presented in an intelligible fashion and written in standard English?

PLOS ONE does not copyedit accepted manuscripts, so the language in submitted articles must be clear, correct, and unambiguous. Any typographical or grammatical errors should be corrected at revision, so please note any specific errors here.

Reviewer #1: Yes

Reviewer #2: Yes

**********

5. Review Comments to the Author

Please use the space provided to explain your answers to the questions above. You may also include additional comments for the author, including concerns about dual publication, research ethics, or publication ethics. (Please upload your review as an attachment if it exceeds 20,000 characters)

**Reviewer #1:**  This is a multisite, prospective, randomized crossover study aimed to identify patient-reported, performance-based, and biomechanical parameters sensitive to ankle-foot device type, providing a foundation for more objective and individualized prescription practices. This is a well designed hypothesis driven summary of the findings. It actually involves the clinical and statistical sensitivity to the devices being examined.

There are several issues requiring some clarification:

1. The Statistical design is presented in the supplemental protocol analysis section. The unit differences generating the power considerations for the specific aims, which are presumably at the alpha-0.05 level and two sided, are not entirely clear. Are these the means or effect size differences expected? Also, they appear a bit large and some motivation would be helpful. There is a power table in the protocol, but the actual connect of the 120 and 30 to the specific aims is not all that clear.

2. Pairwise comparisons are noted in supplemental Tables S1, S2 and S4 and the separation units of 3 and 6 are difficult to picture seeing some of the significant results.

The statistical analysis is routine and what one would expect for this type of endeavor. The analyses involve, descriptives including means, min, max, SD and CV, linear mixed models using the completed data, and linear discriminant analysis , LDA, for group separation.. Bonferroni is used for multiple comparison adjustments. The LDA for key gait features is nicely presented and covariates are incorporated into the analysis. The results are seen on Figures 5 and 6 with key features noted by the coefficients with largest absolute magnitude. All tables and Figures are well formatted and interpretable.

3. For the prediction model, where are the ROC’s and AUC’s since one mentions the logistic regression model in the protocol or did this reviewer miss that somewhere?

As noted this is a well written and ,in addition, an important report. The statistical input was well thought out and executed. Some clarifications ,as noted, would be helpful.

**Reviewer #2:**  The aim of this multisite, prospective, randomized crossover study was to identify patient-reported, performance-based, and biomechanical parameters sensitive to ankle-foot device type, providing a foundation for more objective and individualised prescription practices.

The manuscript describes a technically sound scientific research with data that supports the conclusions. The author test rigoursly 91 individuals biomechanically and with a questioner. The conclusions are drawn appropriately based on the data presented.

The statistical analysis has been performed appropriately and rigorously. All data will be made available upon acceptance of paper. The english is good, at least better than mine.

**********

6. PLOS authors have the option to publish the peer review history of their article (what does this mean?). If published, this will include your full peer review and any attached files.

If you choose “no”, your identity will remain anonymous but your review may still be made public.

**Do you want your identity to be public for this peer review?**  For information about this choice, including consent withdrawal, please see our Privacy Policy.

Reviewer #1: No

Reviewer #2: No

**********

[NOTE: If reviewer comments were submitted as an attachment file, they will be attached to this email and accessible via the submission site. Please log into your account, locate the manuscript record, and check for the action link "View Attachments". If this link does not appear, there are no attachment files.]

To ensure your figures meet our technical requirements, please review our figure guidelines: https://journals.plos.org/plosone/s/figures

You may also use PLOS’s free figure tool, NAAS, to help you prepare publication quality figures: https://journals.plos.org/plosone/s/figures#loc-tools-for-figure-preparation.

NAAS will assess whether your figures meet our technical requirements by comparing each figure against our figure specifications.

# Author comment: Author response to Decision Letter 1

**DOI:** 10.1371/journal.pone.0352644.r002

19 May 2026

Response to Journal Requirement 1: Please ensure that your manuscript meets PLOS ONE's style requirements, including those for file naming.

The manuscript has been revised to meet PLOS ONE style requirements, including the title page and file naming conventions. Specifically, the title page was reviewed against the PLOS ONE formatting templates. Two corrections were made to the affiliations: "United States" in Affiliation 9 was standardized to "USA" to match all other affiliations, and a missing comma was added to Affiliation 6 ("Washington, DC, USA"). All other formatting elements, including author byline symbols, affiliation ordering, corresponding author designation, and consortia notation were confirmed to be compliant.

Response to Journal Requirement 2: Ensure that your code is shared in a way that follows best practice and facilitates reproducibility and reuse.

The statistical analysis code generated by the authors has been deposited in a publicly accessible repository on GitHub (https://github.com/uconn-scs/prescription_study) and archived on Zenodo (DOI: 10.5281/zenodo.20215416) along with the study dataset. The repository is freely accessible and is organized to facilitate reproducibility and reuse in accordance with PLOS ONE's code sharing guidelines. The Data Availability Statement has been updated to reflect this.

Response to Journal Requirement 3: Please provide an amended statement that declares *all* the funding or sources of support (whether external or internal to your organization) received during this study. Please also include the statement “There was no additional external funding received for this study.” in your updated Funding Statement. Please include your amended Funding Statement within your cover letter. We will change the online submission form on your behalf.

The Funding Statement has been amended as requested, following PLOS journal guidelines. The updated statement reads:

"Support was provided by the Congressionally Directed Medical Research Programs–Orthotics and Prosthetics Outcomes Research Program (grant W81XWH-17-2-0014; https://cdmrp.health.mil/) to JTM, ALP, SLP, JTH, CLD, and LMN. There was no additional external funding received for this study. The funders had no role in study design, data collection and analysis, decision to publish, or preparation of the manuscript."

Response to Journal Requirements 4 and 5: Your entire minimal dataset will need to be made freely accessible if your manuscript is accepted for publication.

We acknowledge the journal's recommendation to deposit data prior to acceptance and have done so. All data are fully de-identified. All relevant data and code used to generate tables, plots, and perform statistical analyses in this manuscript are available on GitHub (https://github.com/uconn-scs/prescription_study) and have been archived on Zenodo (DOI: 10.5281/zenodo.20215416). The repository is freely accessible. The Data Availability Statement in the manuscript has been updated to reflect this.

Response to Journal Requirement 6: Please include captions for your Supporting Information files at the end of your manuscript, and update any in-text citations to match accordingly.

Supporting information captions have been added to the end of the manuscript as required. The captions are as follows:

S1 Protocol. Initial institutional review board-approved protocol.

S1 Table. Performance-based outcomes for each ankle-foot device type. Linear mixed model estimates and paired comparisons are shown for the 6-minute walk test (6MWT), Amputee Mobility Predictor (AmpPro), Timed Up and Go test (TUG), Four Square Step Test (4SST), Stair Assessment Index (SAI), and Hill Assessment Index (HAI) measures.

S2 Table. Patient-reported outcomes for each ankle-foot device type. Linear mixed model estimates (SE) and paired comparisons are shown for the 12-Item Short Form Health Survey (SF-12), Prosthesis Evaluation Questionnaire (PEQ), and Orthotics and Prosthetics Users' Survey (OPUS) measures.

S3 Table. Demographics of the biomechanical subset. Participant characteristics are shown as mean (SD) for continuous variables and n (%) for categorical variables.

S4 Table. Biomechanical outcomes across ankle-foot devices at each speed. Linear mixed model estimates (SE) and paired comparisons are shown for the 19 unique biomechanical parameters at the ankle, knee, and hip across the 3 walking speeds for the subset of 29 participants. Percent change from control indicates differences relative to control values. The final column identifies which device had the lowest percent change from control for each parameter. Units are included where relevant.

In-text citations have been reviewed and confirmed to match the supporting information labels above.

Response to Journal Requirement 7: If the reviewer comments include a recommendation to cite specific previously published works, please review and evaluate these publications to determine whether they are relevant and should be cited. There is no requirement to cite these works unless the editor has indicated otherwise.

No reviewer comments included recommendations to cite specific previously published works. Therefore, no action was required for this item.

Response to Journal Requirement 8: Please review your reference list to ensure that it is complete and correct.

The reference list was thoroughly reviewed for completeness, accuracy, and retraction status. Each reference was verified through PubMed and Google Scholar. No retracted articles were identified. All references have been confirmed as current and accurate.

Response to Reviewer #1:

We thank the reviewer for their careful reading of the manuscript and their overall positive assessment of the study design, statistical approach, and presentation. We note that all three comments appear to reference the S1 Protocol, which is the initial IRB-approved protocol submitted as a required supplement per PLOS ONE's clinical trial reporting guidelines. This document was written prior to study initiation as part of the IRB approval process. While it describes the comprehensive data analysis plan for the entire study across all specific aims, it is a historical document and cannot be modified as part of this revision.

We would like to clarify the relationship between the S1 Protocol and this manuscript. This manuscript represents one of several planned publications from a larger, multi-aim study. The S1 Protocol describes the full scope of the study, including analyses planned at study inception that may be addressed in future manuscripts. As is common in longitudinal, multisite clinical trials, the analytical approach evolved over the course of the study in response to the data and findings as they emerged. The statistical analyses reported in this manuscript are specific to the outcomes presented here and are described in full detail in the Statistical Analyses section of the manuscript.

We address each comment individually below:

Comment 1: The reviewer asked about the power considerations, unit differences, and the connection of sample sizes of 120 and 30 to the specific aims as described in the S1 Protocol. These power calculations were developed at study inception to support IRB approval across all specific aims of the larger study. They are not directly relevant to the analyses reported in this manuscript, which uses linear mixed-effects models applied to all available data rather than the power-based sample size targets described in the protocol. The statistical design, including covariates, random effects structure, model assumptions, and multiple comparison adjustments, is described in full in the Statistical Analyses section of this manuscript and is specific to the outcomes reported here.

Comment 2: The reviewer noted that the separation units of 3 and 6 referenced in the S1 Protocol are difficult to interpret in the context of the pairwise comparisons reported in Tables S1, S2, and S4. We clarify that the separation units of 3 and 6 in the protocol refer to power calculation assumptions made at study inception for specific aims of the larger study and have no bearing on the analyses or results reported in this manuscript. The pairwise comparisons presented in the supplemental tables are derived from the linear mixed-effects models described in the Statistical Analyses section, with Tukey's Highest Significant Difference used for multiple comparison adjustment. These results stand independently of the protocol's power assumptions.

Comment 3: The reviewer asked about ROC curves and AUC values in the context of a logistic regression prediction model referenced in the S1 Protocol. We clarify that prediction modeling is not performed in this manuscript and is outside the scope of the current article. This manuscript focuses on identifying performance-based, patient-reported, and biomechanical parameters sensitive to ankle-foot device type. Linear discriminant analysis was used for dimensionality reduction and to identify discriminative gait features, as described in the Statistical Analyses section and presented in Figures 5 and 6. Prediction modeling, including logistic regression and associated metrics such as ROC curves and AUC, will be addressed in a future manuscript as part of the larger dissemination plan for this study. To clarify this for readers, we have added the following sentence to the Discussion: “Additionally, prediction modeling analyses aimed at identifying the strongest independent predictors of optimal prosthetic prescription outcomes will be addressed in future work as part of the broader dissemination plan for this study.”

Response to Reviewer #2

We thank Reviewer 2 for their careful reading of the manuscript and their positive assessment of the study design, statistical approach, and conclusions.

Attachment Submitted filename: Response to Reviewers.docx

# Peer review: Decision Letter 1

**DOI:** 10.1371/journal.pone.0352644.r003

**Authors:** Simone Ranaldi

14 Jun 2026

Toward evidence-based prescription of prosthetic ankle-foot devices: A multisite randomized crossover trial identifying performance-based, patient-reported, and biomechanical parameters sensitive to device type

PONE-D-26-03461R1

Dear Dr. Maikos,

We’re pleased to inform you that your manuscript has been judged scientifically suitable for publication and will be formally accepted for publication once it meets all outstanding technical requirements.

Within one week, you’ll receive an e-mail detailing the required amendments. When these have been addressed, you’ll receive a formal acceptance letter and your manuscript will be scheduled for publication.

An invoice will be generated when your article is formally accepted. Please note, if your institution has a publishing partnership with PLOS and your article meets the relevant criteria, all or part of your publication costs will be covered. Please make sure your user information is up-to-date by logging into Editorial Manager at Editorial Manager® and clicking the ‘Update My Information' link at the top of the page. For questions related to billing, please contact billing support.

If your institution or institutions have a press office, please notify them about your upcoming paper to help maximize its impact. If they’ll be preparing press materials, please inform our press team as soon as possible -- no later than 48 hours after receiving the formal acceptance. Your manuscript will remain under strict press embargo until 2 pm Eastern Time on the date of publication. For more information, please contact onepress@plos.org.

Kind regards,

Simone Ranaldi, Ph.D.

Academic Editor

PLOS One

Additional Editor Comments (optional):

Reviewers' comments:

Reviewer's Responses to Questions

**Comments to the Author**

1. If the authors have adequately addressed your comments raised in a previous round of review and you feel that this manuscript is now acceptable for publication, you may indicate that here to bypass the “Comments to the Author” section, enter your conflict of interest statement in the “Confidential to Editor” section, and submit your "Accept" recommendation.

Reviewer #1: All comments have been addressed

**********

2. Is the manuscript technically sound, and do the data support the conclusions?

The manuscript must describe a technically sound piece of scientific research with data that supports the conclusions. Experiments must have been conducted rigorously, with appropriate controls, replication, and sample sizes. The conclusions must be drawn appropriately based on the data presented.

Reviewer #1: (No Response)

**********

3. Has the statistical analysis been performed appropriately and rigorously?

Reviewer #1: (No Response)

**********

4. Have the authors made all data underlying the findings in their manuscript fully available?

The PLOS Data policy requires authors to make all data underlying the findings described in their manuscript fully available without restriction, with rare exception (please refer to the Data Availability Statement in the manuscript PDF file). The data should be provided as part of the manuscript or its supporting information, or deposited to a public repository. For example, in addition to summary statistics, the data points behind means, medians and variance measures should be available. If there are restrictions on publicly sharing data—e.g. participant privacy or use of data from a third party—those must be specified.

Reviewer #1: (No Response)

**********

5. Is the manuscript presented in an intelligible fashion and written in standard English?

PLOS ONE does not copyedit accepted manuscripts, so the language in submitted articles must be clear, correct, and unambiguous. Any typographical or grammatical errors should be corrected at revision, so please note any specific errors here.

Reviewer #1: (No Response)

**********

6. Review Comments to the Author

Please use the space provided to explain your answers to the questions above. You may also include additional comments for the author, including concerns about dual publication, research ethics, or publication ethics. (Please upload your review as an attachment if it exceeds 20,000 characters)

Reviewer #1: This reviewer was looking at sample size issues from the perspective of the larger study which the investigators have clarified as not applying to this current effort. The remainder of the manuscript was well written and presented according to the analysis performed and the results reported.

**********

7. PLOS authors have the option to publish the peer review history of their article (what does this mean?). If published, this will include your full peer review and any attached files.

If you choose “no”, your identity will remain anonymous but your review may still be made public.

**Do you want your identity to be public for this peer review?**  For information about this choice, including consent withdrawal, please see our Privacy Policy.

Reviewer #1: No

**********

# Editor report: Acceptance letter

**DOI:** 10.1371/journal.pone.0352644.r004

**Authors:** Simone Ranaldi

PONE-D-26-03461R1

PLOS One

Dear Dr. Maikos,

I'm pleased to inform you that your manuscript has been deemed suitable for publication in PLOS One. Congratulations! Your manuscript is now being handed over to our production team.

At this stage, our production department will prepare your paper for publication. This includes ensuring the following:

* All references, tables, and figures are properly cited

* All relevant supporting information is included in the manuscript submission,

* There are no issues that prevent the paper from being properly typeset

You will receive further instructions from the production team, including instructions on how to review your proof when it is ready. Please keep in mind that we are working through a large volume of accepted articles, so please give us a few days to review your paper and let you know the next and final steps.

Lastly, if your institution or institutions have a press office, please let them know about your upcoming paper now to help maximize its impact. If they'll be preparing press materials, please inform our press team within the next 48 hours. Your manuscript will remain under strict press embargo until 2 pm Eastern Time on the date of publication. For more information, please contact onepress@plos.org.

You will receive an invoice from PLOS for your publication fee after your manuscript has reached the completed accept phase. If you receive an email requesting payment before acceptance or for any other service, this may be a phishing scheme. Learn how to identify phishing emails and protect your accounts at https://explore.plos.org/phishing.

If we can help with anything else, please email us at customercare@plos.org.

Thank you for submitting your work to PLOS ONE and supporting open access.

Kind regards,

PLOS ONE Editorial Office Staff

on behalf of

Dr. Simone Ranaldi

Academic Editor

PLOS One
